# Landscape of somatic mutations in different subtypes of advanced breast cancer with circulating tumor DNA analysis

**DOI:** 10.1038/s41598-017-06327-4

**Published:** 2017-07-20

**Authors:** Zongbi Yi, Fei Ma, Chunxiao Li, Rongrong Chen, Lifang Yuan, Xiaoying Sun, Xiuwen Guan, Lixi Li, Binliang Liu, Yanfang Guan, Haili Qian, Binghe Xu

**Affiliations:** 1 Department of Medical Oncology, National Cancer Center/Cancer hospital, Chinese Academy of Medical Sciences and Peking Union Medical College, Beijing, 100021 China; 2 State Key Laboratory of Molecular Oncology, Cancer Institute/Hospital, Chinese Academy of Medical Sciences and Peking Union Medical College, Beijing, 100021 China; 3 Geneplus-Beijing, Beijing, 102206 China; 4 Department of Medical Oncology, Huanxing Cancer Hospital, Beijing, 100005 China

## Abstract

It is particularly important to provide precise therapies and understand tumor heterogeneity based on the molecular typing of mutational landscape. However, the landscape of somatic mutations in different subtypes of advanced breast cancer (ABC) is largely unknown. We applied target-region capture deep sequencing to determine the frequency and spectrum of common cancer-related gene mutations in circulating tumor DNA (ctDNA) among different ABC subtypes and analyze their association with clinical features. In this retrospective study of 100 female advanced breast cancer patients, 96 (96.0%) had somatic genomic alterations in ctDNA, including copy number variants and point mutations. The results revealed that different subtypes of ABC have distinct features in terms of genetic alterations. Multivariate regression analyses revealed that the number of somatic mutations increased with the line of endocrine therapy and the fractions of trunk mutations was positive associated with the line of target therapy.

## Introduction

Breast cancer, one of the most common cancers worldwide, is a heterogeneous disease with a variety of outcomes and drug responses. The St. Gallen subtype classification, introduced in 2011, categorizes breast cancer into five basic therapeutic groups based on immunohistochemical staining. These subtypes include luminal A, luminal B HER2-neu negative, luminal B HER2-neu positive, HER2-neu non-luminal and basal-like. In most cases, the St. Gallen intrinsic subtype classification for breast cancer can effectively predict disease features, recurrence patterns and disease-free survival^1^. However, the outcomes and drug responses of patients with the same subtype are diverse. With the rapid development of genome sequencing technology, molecular characterization based on genomic alteration is widely accepted as a relevant source of cancer stratification. Therefore, it is particularly important to provide precise therapies and understand tumor heterogeneity based on the molecular typing of genomic alteration.

Most molecular studies of breast cancer have focused only on revealing the characteristics of primary cancer based on tissue sequencing^2–4^. As a result of therapeutic selective pressure and tumor evolution, the mutation landscape of advanced breast cancer (ABC) may shift and vary. We need to acquire tissue from the primary tumor and all of the metastatic tumors to understand the mutation characteristics of ABC. However, tissue biopsies are limited by the presence of spatial heterogeneity, which leads to tumor sampling bias^5, 6^. Most of the time, it is difficult to obtain metastatic tissue in clinical work. Circulating tumor DNA (ctDNA) has increasingly attracted attention for its convenient, easily accepted, and minimally invasive method of collection^7^. ctDNA analysis facilitates studies of tumor heterogeneity, for it is able to detect contributions from multiple tumor deposits. A recent study illustrated that ctDNA could reveal tumor heterogeneity in non-small-cell lung cancer^8^.

Here, we applied target-region-capture deep sequencing to detect somatic mutations in plasma ctDNA from ABC patients to understand the mutational characteristics of ABC and analyze the association of clinical features and therapeutic history with gene variations.

## Results

### Samples and clinical data

The main characteristics of these patients are shown in Table 1. A total of 100 female ABC patients were enrolled in the present study. The mean age at breast cancer diagnosis was 45.3 years and all of them are Chinese. One hundred plasma samples collected from these patients were assayed for somatic genomic alterations by target-capture NGS. A panel of 1021 genes was assayed in the present study (Supplementary Table S1).

**Table 1:** Population characteristics

Characteristics	Molecular subtype				All (n = 100)
	HR+/HER2− (n = 28)	HR+/HER2+ (n = 37)	HR− /HER2+ (n = 31)	TNBC (n = 4)	
Average age at diagnosis, y	47.1	42	47.4	44.8	45.3
Age at diagnosis, No. (%)					
<30 y	0 (0.0)	6 (16.2)	0 (0.0)	1 (25.0)	7 (7.0)
30–39 y	8 (28.6)	6 (16.2)	6 (19.4)	1 (25.0)	21 (21.0)
40–49 y	12 (42.9)	18 (48.6)	11 (35.5)	1 (25.0)	42 (42.0)
50–59 y	4 (14.3)	6 (16.2)	11 (35.5)	0 (0.0)	21 (21.0)
≥60	4 (14.3)	1 (2.7)	3 (9.7)	1 (25.0)	9 (9.0)
Menstruation status, No. (%)					
Premenopausal	19 (67.9)	28 (75.7)	18 (58.1)	3 (75.0)	68 (68.0)
Postmenopausal	9 (32.1)	9 (24.3)	13 (41.9)	1 (25.0)	32 (32.0)
Tumor stage at first diagnosis, No. (%)					
I	3 (10.7)	4 (10.8)	4 (12.9)	1 (25.0)	12 (12.0)
II	12 (42.9)	12 (32.4)	12 (38.7)	2 (50.0)	38 (38.0)
III	7 (25.0)	10 (27.0)	11 (35.5)	1 (25.0)	29 (29.0)
IV	6 (21.4)	11 (29.7)	4 (12.9)	0 (0.0)	21 (21.0)
Tumor size, No. (%)					
T1	8 (28.6)	13 (35.1)	11 (35.5)	1 (25.0)	33 (33.0)
T2	19 (67.9)	17 (45.9)	17 (54.8)	3 (75.0)	56 (56.0)
T3	1 (3.6)	5 (13.5)	3 (9.7)	0 (0.0)	9 (9.0)
T4	0 (0.0)	2 (5.4)	0 (0.0)	0 (0.0)	2 (2.0)
Number of positive nodes, No. (%)					
0	8 (28.6)	7 (18.9)	8 (25.8)	1 (25.0)	24 (24.0)
1–3	8 (28.6)	9 (24.3)	8 (25.8)	2 (50.0)	27 (27.0)
4–9	8 (28.6)	11 (29.7)	8 (25.8)	0 (0.0)	27 (27.0)
>9	4 (14.3)	10 (27.0)	7 (22.6)	1 (25.0)	22 (22.0)
Presence or absence of metastasis at first diagnosis, No. (%)					
Presence	6 (21.4)	11 (29.7)	4 (12.9)	0 (0.0)	21 (21.0)
Absence	22 (78.6)	26 (70.3)	27 (87.1)	4 (100.0)	79 (79.0)
Nuclear grade, No. (%)					
1	0 (0.0)	1 (2.7)	3 (9.7)	0 (0.0)	4 (4.0)
2	15 (53.6)	25 (67.6)	15 (48.4)	2 (50.0)	57 (57.0)
3	13 (46.4)	11 (29.7)	13 (41.9)	2 (50.0)	39 (39.0)
Number of metastatic sites, No. (%)					
1	4 (14.3)	8 (21.6)	14 (45.2)	2 (50.0)	28 (28.0)
2–3	14 (50.0)	26 (70.3)	15 (48.4)	1 (25.0)	56 (56.0)
≥4	10 (35.7)	3 (8.1)	2 (6.5)	1 (25.0)	16 (16.0)
Line of chemotherapy, No. (%)					
0	3 (10.7)	1 (2.7)	2 (6.5)	1 (25.0)	7 (7.0)
1	11 (39.3)	11 (29.7)	14 (45.2)	1 (25.0)	37 (37.0)
≥2	14 (50.0)	25 (67.6)	15 (48.4)	2 (50.0)	56 (56.0)
Line of endocrine therapy, No. (%)					
0	1 (3.6)	6 (16.2)	31 (100.0)	4 (100.0)	42 (42.0)
1	5 (17.9)	15 (40.5)	0 (0.0)	0 (0.0)	20 (20.0)
≥2	22 (78.6)	16 (43.2)	0 (0.0)	0 (0.0)	38 (38.0)
Line of anti-HER2 therapy, No. (%)					
0	28 (100.0)	12 (32.4)	10 (32.3)	4 (100.0)	54 (54.0)
≥1	0 (0.0)	25 (67.6)	21 (67.7)	0 (0.0)	46 (46.0)

HR hormone receptor, HER2 human epidermal growth factor 2,TNBC triple-negative breast cancer.

### Identification of somatic genomic alterations

**Figure 1:**
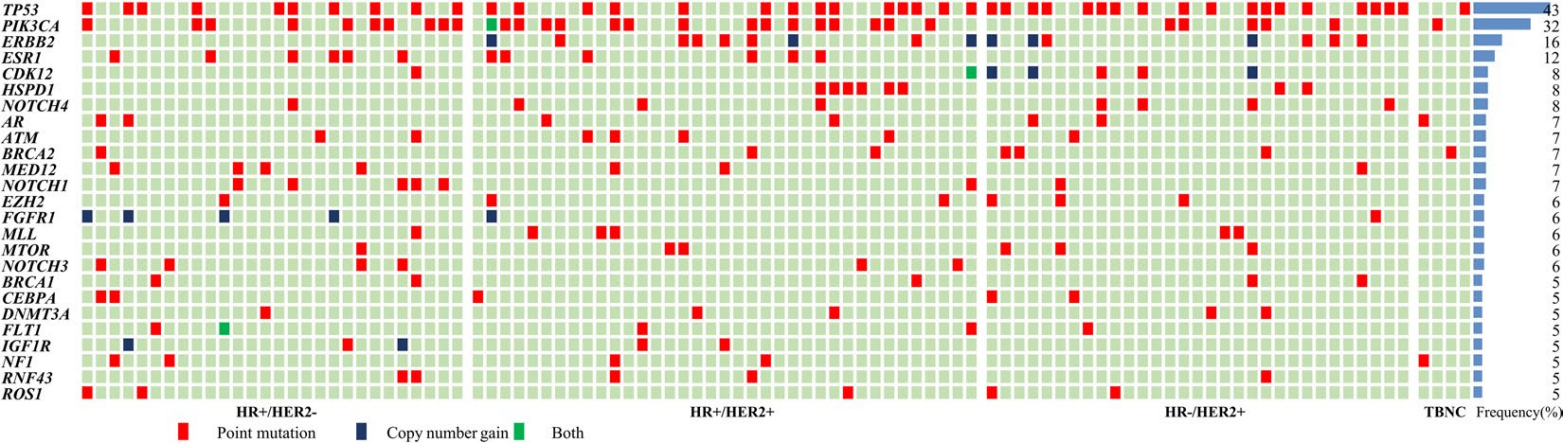
The landscape of hotspot mutations in advanced breast cancer. Each of the 25 hotspot gene mutations has been identified in more than 5 patients listed to the left of the figure. The number of mutations in each gene among the 100 patients is shown (rows). Point mutations, copy number changes and both are colored red, blue and purple, respectively.

Somatic genomic alterations in ctDNA, including CNVs and point mutations, were identified in 96 of 100 patients (96.0%). (Figure 1)

Target-capture sequencing of plasma ctDNA and matched WBC DNA was performed to detect somatic mutations in each sample, achieving a mean sequencing coverage of 1130×(317~3015×) for plasma ctDNA and 317×(69~541×) for WBC DNA. The sequencing and data analysis pipline used in this study were validated and the results showed that our test had 96.30% sensitivity at mutant allele frequency (MAF)≥0.5% with high specificity (99.9997%) and accuracy (99.9996%) for SNV detection. For CNV detection, the approach had 95.83% sensitivity for copy numbers at 1.25×(25.6% extra copies) with high specificity (99.77%) and accuracy (99.76%)^9^. In additional, the final candidate somatic variations were all manually verified in IGV browser.

**Figure 2:**
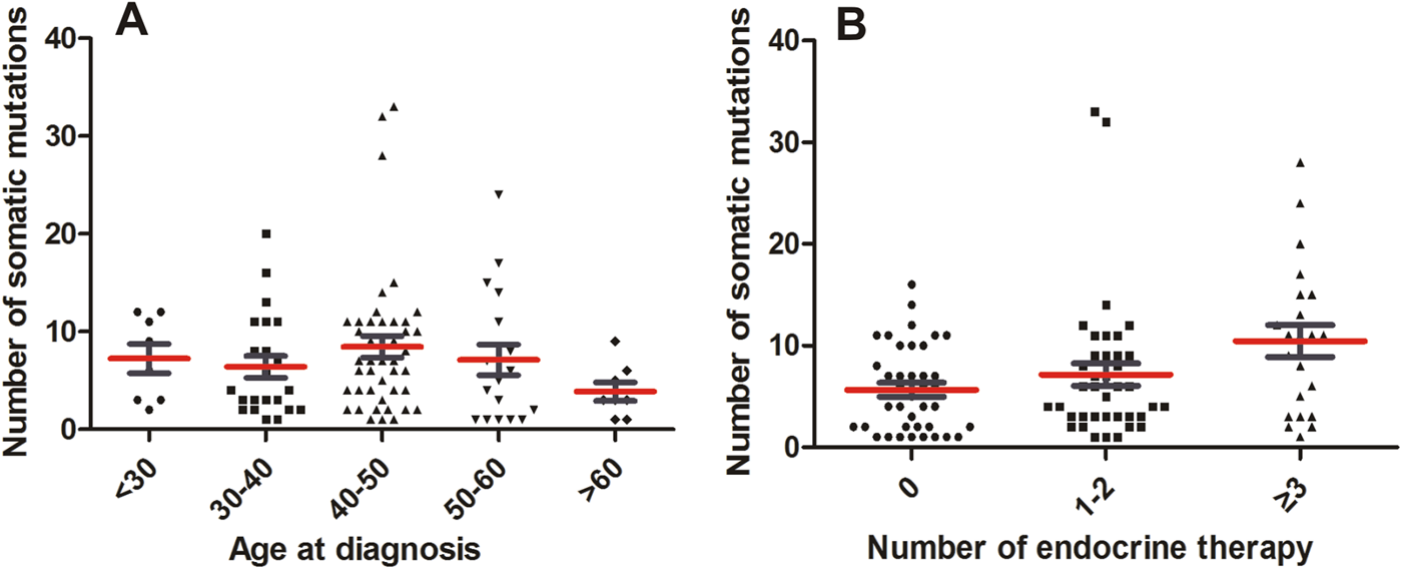
The correlation between the number of somatic mutations and clinical characteristics. (**A**).The relationship between the number of somatic mutations and the age at diagnosis in all 100 patients. (**B**).The correlation between the number of somatic mutations and the line of endocrine therapy in all 100 patients. Dots correspond to number of somatic mutations and whiskers correspond to its Standard Error. Red line correspond to the mean number of somatic mutations.

The number of somatic mutations varied markedly between individual patients (mean 2.9, range 1–31). No difference in the number of somatic mutations was found between the four examined subtypes (*p* > 0.05). We examined the relationship between the number of somatic mutations and the age at diagnosis in the 100 patients. Across the entire sample and within each of the four subtype groups, no correlation was observed between the number of somatic mutations and the patient’s age. However, the mean number of somatic mutations was higher in patients aged 40–50 years than in patients aged more than 60 years (8.46 vs 3.88, respectively; *p* = 0.039). The results of multivariate analyses showed that the number of somatic mutations increased with the line of endocrine therapy (*p* = 0.007, Table 2). However, there were no differences between the number of mutations and the line of chemotherapy or target therapy (*p* > 0.05) (Fig. 2).

**Table 2:** Multivariate analyses of the associations between the number of somatic mutations and clinical characteristics.

	*B*	*t*	*p*	*95% CI*	
Number of positive nodes at first diagnosis	−1.030	−1.377	0.172	−2.514	0.455
Tumor stage at first diagnosis	1.552	1.823	0.071	−0.138	3.242
Line of endocrine therapy	1.156	2.772	0.007	0.328	1.983

The fractions of trunk mutations were ranged from 0.3–80%. Multivariate regression analyses revealed that the fractions of trunk mutations was positive associated with the line of target therapy (*p* = *0.035*, Table 3).

**Table 3:** Multivariate analyses of the associations between the fractions of trunk mutations and clinical characteristics.

	*B*	*t*	*p*	*95% CI*	
Menstruation status	5.598	1.602	0.113	−1.341	12.536
ki-67	−0.121	−1.517	0.133	−0.279	0.037
Number of metastatic sites	0.716	0.516	0.607	−2.039	3.471
Line of target therapy	3.28	2.133	0.035	0.227	6.332

### CNVs

We identified CNVs in 13 out of 100 (13.0%) patients by analyzing the sequencing data for plasma matched with blood cells from the same patient (Supplementary Table S2). Thirteen genes had copy number gains. Amplification of the *ERBB2* gene, which encodes the HER2 protein, was predominant and was identified in 6 of 100 (6.0%) patients, all of whom belonged to the HER2+ group. Amplification of the *FGFR1* gene was also identified in 5 of 100 (5.0%) patients, all of whom belonged to the HR+ group. Elevated levels of *CDK12* were present in 4 of 100 patients (4.0%), all of whom were characterized by *ERBB2* and *CDK12* co-amplification. Moreover, amplification of the *AURKA*, *IGFR1* and *RPS6KB1* genes was captured in 2 of 100 patients (2.0%). In addition, ctDNA sequencing identified 7 other, less common CNVs in the study population; each of these CNVs was only detected in 1 patient (1.0%).

### Point mutations

Point mutations in breast cancer-related genes were present in 96 of 100 patients (96.0%, Supplementary Table S3). *TP53* and *PIK3CA* were the two most frequently mutated genes detected in the ctDNA of the 100 patients; these genes appeared in 43 (43.0%) and 32 (32.0%) patients, respectively. *TP53* was the most frequently mutated gene, appearing in 43 (43.0%) patients. Thirty-nine kinds of mutations were detected, and 3 of the patients had *R273H* mutations. *PIK3CA* gene mutations were detected in 32 (32.0%) patients and included 17 point mutations and 1 CNV: *H1047R* mutation was the most frequently detected of these, appearing in 14 patients, while 4 patients had *N345K* mutations, 4 *E542K*, and 3 *E545K*. Twelve patients had *ESR1* mutations, including 7 patients with *Y537S* mutations, 7 with *D538G* mutations and 4 with other kinds of point mutations. *ERBB2* mutations were detected in 11 (11%) patients, all of whom belonged to the HER2-positive group.

### Genomic characterization of the molecular subtypes

We analyzed the CNVs and point mutations of each patient in our cohort to delineate the biological characteristics of each molecular subtype of ABC.

**Figure 3:**
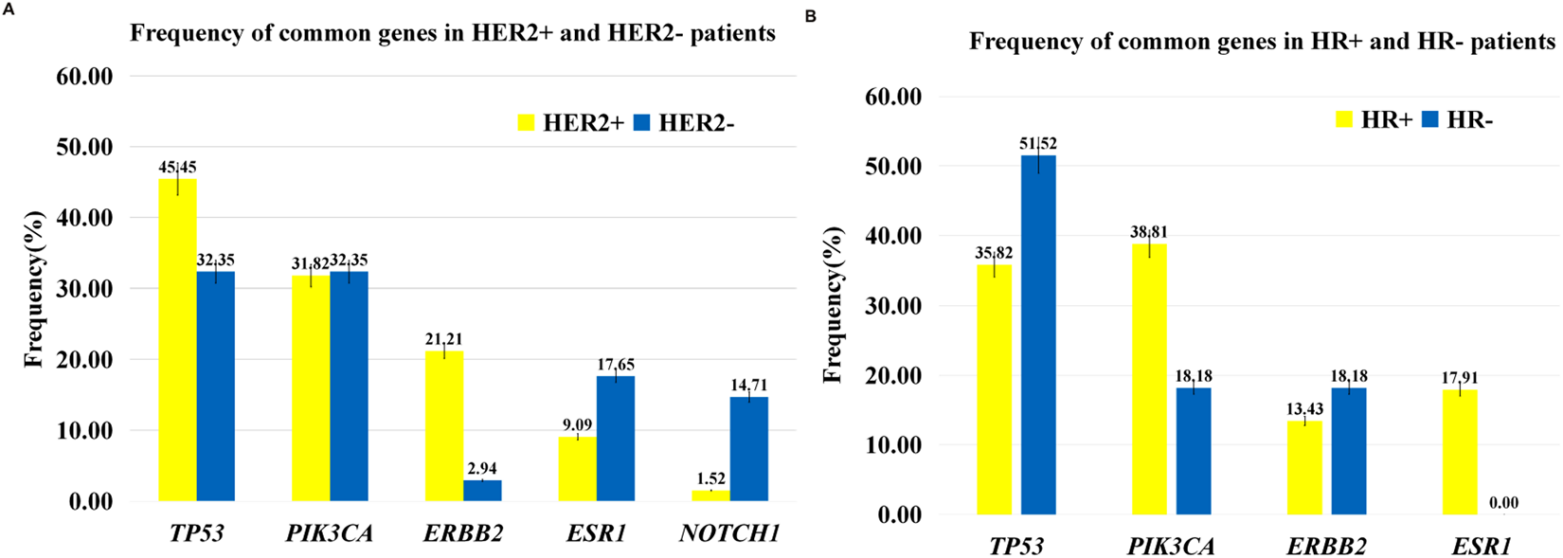
The frequency of common genes in four subtypes among 100 patients. (**A**) The frequency of common genes in different HER2 statues. Four gene mutations including *TP53/PIK3CA/ERBB2/ESR1* detected more than 10 patients and *NOTCH1* which differential detected between different HER2 statues patients listed to the bottom the figure. HER2 positive and negative patients are colored yellow and blue respectively. (**B**) The frequency of common genes in different HR statues patients. Four gene mutations including *TP53/PIK3CA/ERBB2/ESR1* detected more than 10 patients listed to the bottom of the figure. HR positive and negative patients are colored yellow and blue respectively. Whiskers correspond to the percentage Standard Error of the gene frequency.

There were 28 patients (28.0%) in the HR-positive HER2-negative group. We detected 8 gene CNVs in 7 of these 28 patients (25.0%). The frequency of mutations, including CNVs and point mutations, of eight genes was more than 10% in all 28 patients. The HR-positive HER2-positive group contained 37 patients (37.0%). We detected 6 genes whose mutation frequency was more than 10%, and 4 gene CNVs were detected in 3 patients (8.1%) from this group. Ten genes were had variations of more than 10% in the HR-negative HER2-positive group, which contained 31 patients (31.0%). Three patients (9.7%) out of 31 had CNVs. The triple-negative breast cancer (TNBC) group only contained four patients (4.0%), and 22 gene mutations were detected. ESR1 mutations were detected in 12 of the HR+ patients, who had a significantly higher mutation frequency than did the HR− patients (18.46% vs 0.00%, respectively; *p* = 0.007). *NOTCH1* mutations were detected in 5 of the HER2- patients, which was more than the number of HER2+ patients that had such mutations (15.63% vs 2.94%, respectively; *p* = 0.033). Mutations in three genes, namely, *NOTCH3, ESR1* and *FGFR1*, were detected in only the HR-positive group. *PIK3CA* mutations occurred more frequently in HR+ patients than in HR− patients (40.00% vs 17.14%, respectively; *p* = 0.025) (Fig. 3).

### The associations between point mutations and clinical characteristics

Multiple logistic regression analysis indicated that pathological grade, tumor size at diagnosis and PR status were positively associated with *PIK3CA* mutations. However, menstruation status was inversely associated with *PIK3CA* mutations. Multiple regression analysis also revealed that ki-67, the presence of metastasis at diagnosis, the number of metastatic sites, and the line of endocrine therapy were associated with *ESR1* mutations. HER2 status, number of metastatic sites and tumor size at diagnosis were negatively associated with *NOTCH1* mutations in multiple regression analysis. (Table 4).

**Table 4:** Multivariate analyses of the associations between somatic mutations and clinical characteristics.

	*p*	*OR*	*95% CI*	
***PIK3CA***				
Pathological grade	0.006	3.904	1.488	10.244
Tumor size	0.031	2.224	1.076	4.597
Menstruation status	0.036	0.279	0.085	0.918
PR status	0.020	3.382	1.208	9.469
***ESR1***				
ki-67	0.042	1.043	1.001	1.086
Presence or absence of metastasis at first diagnosis	0.018	7.679	1.410	41.830
Number of metastatic sites	0.043	1.990	1.023	3.872
Line of endocrine therapy	0.008	2.133	1.221	3.728
Pathological grade	0.525	1.661	0.347	7.945
Line of target therapy	0.331	1.340	0.743	2.419
***NOTCH1***				
HER2 status	0.019	0.047	0.004	0.601
Tumor size	0.013	0.029	0.002	0.468
Number of metastatic sites	0.020	0.133	0.024	0.725
PR status	0.057	18.187	0.918	360.49

PR progesterone receptor, HER2 human epidermal growth factor 2.

## Discussion

Currently, breast cancer is divided into different molecular subtypes based on the St. Gallen subtype classification, which can effectively predict disease features and prognosis^1^. However, the outcomes and drug responses of patients with the same subtype are also diverse. Several studies revealed that one tumor often consists of multiple cell subpopulations and this heterogeneity has been proposed as one of the major reasons for the failure of drug treatment^10, 11^. Gene variations based subtyping has the potential to improve or even replace the current classification system^12, 13^. Several breast cancer studies have revealed mutations characteristic of primary cancer based on tissue sequencing^2–4^. Because of the spatial and temporal heterogeneity of breast cancer, the genetic alterations of ABC are different from those of early breast cancer. This study applied the next-generation sequencing of ctDNA to understand the mutant characterization of different subtypes of ABC and analyze the association between clinical features and gene somatic variation profiling. The results revealed that different subtypes of ABC have distinct features with respect to genetic alterations.

The somatic mutations in a cancer genome accumulate over a patient’s lifetime, and the number of somatic mutations may increase with the age^2^. A previous study found no correlation between the number of somatic mutations and age both in ER+ and ER− breast cancer patients^2^. Our data also support this conclusion, as there was no relationship between the number of somatic mutations and age in any of the four examined subtypes. However, the number of somatic mutations was higher in patients aged 40–50 years than in patients aged more than 60 years. The basis for this pronounced difference is unclear and may be correlated with therapy because multivariate analyses showed that the number of somatic mutations increased with the line of endocrine therapy. Recent evidences suggest that a high degree of cancer cell heterogeneity could lead to drug resistance^10^. The number of somatic mutations increased with the line of endocrine therapy maybe a result of endocrine therapy resistance. But we did not found the same phenomenon for target therapy and chemotherapy and further research is needed on this subject. We also found that the fraction of trunk mutations increased with the line of target therapy. This maybe the result of target therapy selection or maybe a result of drug resistance.

In general, *TP53* and *PIK3CA* are the most frequently mutated genes in ABC, which is consistent with early breast cancer. *TP53*, the most frequently mutated gene in breast cancer, is more frequently mutated in HER2-positive breast cancer and TNBC^14, 15^. We did not find any significant difference in *TP53* mutation frequency among the subtypes of ABC. No relationship was observed between clinical characteristics and *TP53* mutations. *PIK3CA* is the second most frequently mutated gene, following *TP53*, currently known in breast cancer patients, occurring at a frequency of 20–45% in early breast cancer^16–20^. *PIK3CA* mutations are more prevalent in the HR+/HER2+ subtype of breast cancer^19, 20^. The results of the present study suggest that mutations are significantly more frequent in HR+ cancers than in *HR*− cancers^19–21^. The association of *PIK3CA* mutations with HR and HER2 status remains controversial. Some studies have supported a positive association between *PIK3CA* mutations and hormonal and HER2 status, while others have suggested no significant correlation^21–27^. Our data suggest that *PIK3CA* mutations are associated with progesterone receptor status, but there was no association with estrogen receptor or HER2 status. Multivariate analysis indicated that pathological grade and tumor size at diagnosis were positively associated with *PIK3CA* mutations, which indicates that cancers with *PIK3CA* mutations may be more invasive.

Mutations in the ER gene (*ESR1*) have been described in ABCs that had been exposed to previous therapy with aromatase inhibitors (AIs)^28, 29^. *ESR1* mutations are only rarely detectable in primary breast cancer and are only found at an appreciable frequency after the development of hormone resistance^30, 31^. The frequency of *ESR1* mutations ranges from 20% to 89% in HR-positive patients previously exposed to AIs^28–30^. Our study found that the mutation frequency of *ESR1* was significantly higher in HR+ patients than in HR− patients, especially in the HR+/HER2− group. *ESR1* mutations were positively related to ki-67 index, the presence or absence of metastasis at first diagnosis, the number of metastatic sites, and the line of endocrine therapy. These results support the previous view that *ESR1* mutations are acquired from endocrine therapy^28^.

The *NOTCH1* signaling pathway is associated with cell proliferation, cell differentiation, cell motility, cell-cell connections and cell polarity^32^. *NOTCH1* was recently found to be related to cancer cell metastasis and the maintenance of cancer stem cells^33^. A recent meta-analysis found that the expression of *NOTCH1* was enriched in the triple-negative subtype of breast cancer^34^. Previous studies indicated that *NOTCH1* was inversely correlated with HR status, but there was no significant relationship between *NOTCH1* and HER2 status^34^. Our study detected *NOTCH1* gene mutations more frequently in the HR+/HER2− group, in contrast to previous research. Multivariate analysis in the present study found that *NOTCH1* mutations were significantly negatively correlated with HER2 status. We also found that *NOTCH1* mutations were negatively related to the number of metastatic sites. The mechanism is unclear and the result need further study and verify for our limited sample size in *NOTCH1* mutant group.

*ERBB2* point mutations and CNVs were only detected in HER2+ cancers, and the mutant frequency in the HER2+ group was 8.8%. Theoretically, all HER2+ patients should show an *ERBB2* copy number gain in ctDNA. However, only 8.8% patients in the HER2+ group showed an *ERBB2* copy number gain. Although the variation in the timing of primary tissue and metastatic plasma sampling may partly account for the discordance, possible methodological concerns were still explored. The ctDNA assay may not be sufficiently sensitive to accurately detect CNVs.

Despite the advantages delineated above, several limitations of the study should be noted. To begin, HER2 amplification was identified by ctDNA assay in only 6 of 68 (8.8%) HER2+ patients. Further studies should be conducted to improve the sensitivity of the ctDNA assay to detect CNVs. Second, the depth of coverage was low for most of the ctDNA samples; this limitation could be addressed in future studies by improving the sequencing depth. Third, the present study did not have sufficient power to arrive at statistically sound conclusions for the small number of patients with each subtype, especially for the TNBC group.

In conclusion, our data indicate that different subtypes of ABC have distinct features in terms of genetic alterations. Certain gene mutations may be related to clinical treatment, especially endocrine therapy.

## Materials and Methods

### Patients and sample collection

The present study is a retrospective study. All blood samples were obtained from female ABC patients who underwent therapy at Cancer Hospital, Chinese Academy of Medical Sciences, from March 2015 to September 2016. The study group comprised 100 patients with invasive ductal carcinoma, ranging from 22 to 72 years of age. Patients were eligible for enrollment if they were over 18 years of age and had a histologic/cytologic diagnosis of metastatic breast cancer. Patients were excluded if they had received chemotherapy within the past month. The study was reviewed and approved by the ethical committee at National Cancer Center/Cancer hospital, Chinese Academy of Medical Sciences and Peking Union Medical College. All methods were performed in accordance with the relevant guidelines and regulations, and informed consent was obtained from the patients and parents.

### DNA extraction

Circulating DNA was isolated from 0.6–1.8 mL of plasma using the QIAamp Circulating Nucleic Acid Kit (Qiagen) and extracted using the DNeasy Blood and Tissue Kit. The DNA concentration was assessed using a Qubit fluorometer (Invitrogen, Carlsbad, CA USA) and the Qubit dsDNA HS (High Sensitivity) Assay Kit. The size distribution of the cell-free DNA (cfDNA) was assessed using an Agilent 2100 Bioanalyzer and the DNA HS kit (Agilent Technologies, Santa Clara, CA, USA).

### Target capture and next-generation sequencing

Sequencing libraries were prepared for cfDNA using the KAPA DNA Library Preparation Kit (Kapa Biosystems, Wilmington, MA, USA), and gDNA sequencing libraries were prepared using the protocols recommended in the Illumina TruSeq DNA Library Preparation Kit (Illumina, San Diego, CA). For samples at or near the minimum input requirement, additional pre-capture PCR cycles were performed to generate sufficient PCR product for hybridization. Libraries were hybridized to custom-designed biotinylated oligonucleotide probes (Roche NimbleGen, Madison, WI, USA) covering ~1.1 Mbp of sequence. DNA sequencing was carried out with the HiSeq 3000 Sequencing System (Illumina, San Diego, CA) with 2 × 101-bp paired-end reads.

### Sequencing data analysis

After removing the terminal adaptor sequences and low-quality data, the reads were mapped to the reference human genome. GATK (https://www.broadinstitute.org/gatk/, The Genome Analysis Toolkit) and MuTect were used to call small insertions and deletions (indels) and single nucleotide variants (SNVs) in the somatic DNA by filtering peripheral blood (PBL) sequencing data. In addition, we used the NoahCare Tool Kit using NCfilter (software developed by self, version 1.5.0) for fastq data QC, NCbamInfo (version 0.2.0) for alignment QC; NCanno (version 0.1.1) for annotation with multiple databases; and NChot (version 0.1.0) for hotspot region variant review and recall. Contra was used to detect copy number variants, and BreakDancer was used to detect cancer-associated structural variants. The final candidate variants were all manually verified using the Integrative Genomics Viewer (IGV) Browser.

### Statistical analysis

The relationship between the molecular subtypes and gene variation profiles was studied by cross-tabulation with the chi-square test or Fisher’s exact test. Potential links between various parameters and the occurrence of a particular mutation were evaluated using logistic regression. Linear regression models were constructed examining the relationship between mutant allele fractions of trunk mutation or the number of total mutations and clinical characteristics of ABC. Mutations, which had the highest predicted cellular prevalence, were classified as trunk mutations. The Mann-Whitney U was used to compare the mean number of somatic mutations between different age groups. All statistical tests were two-sided, and p < 0.05 was considered to indicate statistical significance. All statistical analyses were performed using SPSS version 19.0 (SPSS Company, Chicago, IL).

## Electronic supplementary material

Supplementary tables

## References

[1] Vasconcelos I (2016). The St. Gallen surrogate classification for breast cancer subtypes successfully predicts tumor presenting features, nodal involvement, recurrence patterns and disease free survival. Breast.

[2] Stephens PJ (2012). The landscape of cancer genes and mutational processes in breast cancer. Nature.

[3] Nik-Zainal S (2016). Landscape of somatic mutations in 560 breast cancer whole-genome sequences. Nature.

[4] Koboldt DC (2012). Comprehensive molecular portraits of human breast tumours. Nature.

[5] Saunders NA (2012). Role of intratumoural heterogeneity in cancer drug resistance: molecular and clinical perspectives. EMBO Mol Med.

[6] Schmitt MW, Loeb LA, Salk JJ (2016). The influence of subclonal resistance mutations on targeted cancer therapy. Nat Rev Clin Oncol.

[7] Crowley E, Di Nicolantonio F, Loupakis F, Bardelli A (2013). Liquid biopsy: monitoring cancer-genetics in the blood. Nat Rev Clin Oncol.

[8] Chabon JJ (2016). Circulating tumour DNA profiling reveals heterogeneity of EGFR inhibitor resistance mechanisms in lung cancer patients. Nature Communications.

[9] Yang, X. *et al*. Technical Validation of a Next-Generation Sequencing Assay for Detecting Clinically Relevant Levels of Breast Cancer-Related Single-Nucleotide Variants and Copy Number Variants Using Simulated Cell-Free DNA. *J Mol Diagn*. doi:10.1016/j.jmoldx.2017.04.007 (In Press). 10.1016/j.jmoldx.2017.04.007 28502728

[10] Wang E (2013). Cancer Systems Biology in the Genome Sequencing Era: Part 2, Evolutionary Dynamics of Tumor Clonal Networks and Drug Resistance. Semin Cancer Biol..

[11] Wang E (2013). Cancer Systems Biology in the Genome Sequencing Era: Part 1, Dissecting and Modeling of Tumor Clones and their Networks. Semin Cancer Biol..

[12] Zaman N (2013). Signaling Network Assessment of Mutations and Copy Number Variations Predict Breast Cancer Subtype-Specific Drug Targets. Cell Rep..

[13] Zou J, Wang E (2017). ETumorType, an Algorithm of Discriminating Cancer Types for Circulating Tumor Cells or Cell-Free DNAs in Blood. Genomics, Proteomics & Bioinformatics..

[14] Ma CX, Reinert T, Chmielewska I, Ellis MJ (2015). Mechanisms of aromatase inhibitor resistance. Nature Reviews Cancer.

[15] Kim J (2016). Association between Mutation and Expression of TP53 as a Potential Prognostic Marker of Triple-Negative Breast Cancer. Cancer Research and Treatment.

[16] Loibl S (2016). PIK3CA mutations are associated with reduced pathological complete response rates in primary HER2-positive breast cancer: pooled analysis of 967 patients from five prospective trials investigating lapatinib and trastuzumab. Ann Oncol.

[17] Arthur LM (2014). Changes in PIK3CA mutation status are not associated with recurrence, metastatic disease or progression in endocrine-treated breast cancer. Breast Cancer Research and Treatment.

[18] Oshiro C (2015). PIK3CA mutations in serum DNA are predictive of recurrence in primary breast cancer patients. Breast Cancer Research and Treatment.

[19] Dirican E, Akkiprik M, Özer A (2016). Mutation distributions and clinical correlations of PIK3CA gene mutations in breast cancer. Tumor Biology.

[20] Arsenic R (2014). Analysis of PIK3CA mutations in breast cancer subtypes. Appl Immunohistochem Mol Morphol.

[21] Ahmad, F., Badwe, A., Verma, G., Bhatia, S. & Das, B.R. Molecular evaluation of PIK3CA gene mutation in breast cancer: determination of frequency, distribution pattern and its association with clinicopathological findings in Indian patients. *Medical Oncology* **33** (2016). 10.1007/s12032-016-0788-y 27282497

[22] Kalinsky K (2009). PIK3CA mutation associates with improved outcome in breast cancer. Clin Cancer Res.

[23] Cizkova M (2012). PIK3CA mutation impact on survival in breast cancer patients and in ERalpha, PR and ERBB2-based subgroups. Breast Cancer Res.

[24] Barbareschi M (2007). Different prognostic roles of mutations in the helical and kinase domains of the PIK3CA gene in breast carcinomas. Clin Cancer Res.

[25] Perez-Tenorio G (2007). PIK3CA mutations and PTEN loss correlate with similar prognostic factors and are not mutually exclusive in breast cancer. Clin Cancer Res.

[26] Ellis MJ (2010). Phosphatidyl-inositol-3-kinase alpha catalytic subunit mutation and response to neoadjuvant endocrine therapy for estrogen receptor positive breast cancer. Breast Cancer Res Treat.

[27] Maruyama N (2007). Clinicopathologic analysis of breast cancers with PIK3CA mutations in Japanese women. Clin Cancer Res.

[28] Schiavon G (2015). Analysis of ESR1 mutation in circulating tumor DNA demonstrates evolution during therapy for metastatic breast cancer. Sci Transl Med.

[29] Jeselsohn R, Buchwalter G, De Angelis C, Brown M, Schiff R (2015). ESR1 mutations-a mechanism for acquired endocrine resistance in breast cancer. Nat Rev Clin Oncol.

[30] Niu J (2015). Incidence and clinical significance of ESR1 mutations in heavily pretreated metastatic breast cancer patients. Onco Targets Ther.

[31] Toy W (2013). ESR1 ligand-binding domain mutations in hormone-resistant breast cancer. Nat Genet.

[32] Andersson ER, Lendahl U (2014). Therapeutic modulation of Notch signalling — are we there yet?. Nature Reviews Drug Discovery.

[33] Hanahan D, Weinberg RA (2011). Hallmarks of cancer: the next generation. Cell.

[34] Yuan X (2015). Expression of Notch1 Correlates with Breast Cancer Progression and Prognosis. PLOS ONE.

