# Supplementary material for: Landscape of somatic mutations in different subtypes of advanced breast cancer with circulating tumor DNA analysis
Source: Sci Rep. 2017 Jul 20;7:5995. doi: 10.1038/s41598-017-06327-4 (PMC5519668; doi:10.1038/s41598-017-06327-4)
Supplement: Supplementary file 1 — Supplementary tables [file 41598_2017_6327_MOESM1_ESM.pdf]

Landscape of somatic mutations in different subtypes of advanced breast cancer with circulating tumor DNA analysis

Zongbi Yi[1], Fei Ma[1#], Chunxiao Li[2], Rongrong Chen[3]， Lifang Yuan[4], Xiaoying Sun[4], Xiuwen Guan[1], Lixi Li[1]， Binliang Liu[1]， Yanfang Guan[3], Haili Qian[2], Binghe Xu[1]

1. Department of Medical Oncology, National Cancer Center/Cancer hospital, Chinese Academy of Medical Sciences and Peking Union Medical College, Beijing, 100021, China
2. State Key Laboratory of Molecular Oncology, Cancer Institute/Hospital, Chinese Academy of Medical Sciences and Peking Union Medical College, Beijing, 100021, China
3. Geneplus-Beijing, Beijing, 102206, China
4. Department of Medical Oncology, Huanxing Cancer Hospital, Beijing, 100005, China

[#]Corresponding Author:

Fei Ma, MD

Department of Medical Oncology

National Cancer Center/Cancer Hospital

Chinese Academy of Medical Sciences and Peking Union Medical College

No.17, Panjiayuan Nanli, Chaoyang District

Beijing 100021, China

Email:drmafei@126.com

Telephone: 8610-87787652

Fax: 8610-87714054

Summary of supplementary tables

Supplementary Table S1. List of target region genes

Supplementary Table S2. Somatic point mutations identified in 100 plasma samples

Supplementary Table S3. Somatic copy number variants (CNVs) identified in 100 plasma samples

Supplementary Table S1. List of target region genes

ABCA10	CSNK1E	HSPD1	NUP210	SLC2A2
ABCA8	CSPP1	HYDIN	NUTM1	SLC30A5
ABCB7	CTCF	IBSP	NWD1	SLC35B2
ABCC8	CTIF	IDH1	NXF1	SLC35B4
ABCF2	CTNNA2	IDH2	NXF5	SLC38A4
ABL1	CTNNB1	IFT172	OBP2A	SLC38A5
ABL2	CTSF	IGF1R	OBP2B	SLC43A1
ACE	CYP2A13	IGSF9	OCA2	SLC45A1
ACER2	CYP3A4	IKBKAP	ODZ3	SLC4A10
ACOT11	CYP4A11	IKBKE	OR2T4	SLC4A4
ACPP	CYTH4	IL11RA	OR4A15	SLC5A1
ACSL1	DCLK2	IL13RA2	OR4C6	SLC6A5
ACSM5	DCST1	IL1RAPL1	OR5L2	SLC8A1
ACSS3	DDB1	IL27RA	OR6F1	SLCO1B7
ACTL6B	DDR1	IL7R	OSBPL10	SLCO5A1
ADAM23	DDR2	IMPG1	OTOA	SMARCA4
ADAM33	DDX24	INHBA	OTOGL	SMARCB1
ADAMTS12	DDX3X	INPP4B	OVCH1	SMO
ADAMTS16	DEPDC4	INPP5J	P4HB	SMTN
ADAMTS19	DGKK	IQCA1	PABPC4	SNTG1
ADAMTS20	DHCR24	IRS2	PACS2	SORCS3
ADAMTS5	DHDDS	ITFG2	PAEP	SPAG16
ADAMTSL1	DHX9	ITGA8	PAGE1	SPATA13
ADD2	DIAPH1	ITGA9	PALB2	SPG20
AGMAT	DKC1	ITIH1	PARK2	SPINT1
AGTPBP1	DLST	ITLN2	PARP4	SPPL2A
AHCTF1	DMD	ITM2A	PCK2	SPPL3
AK5	DMXL1	ITPKB	PCLO	SPRED1
AKR1B10	DMXL2	ITPR1	PCNT	SPTA1
AKR1C1	DNAH10	JAK1	PCNXL2	SRC
AKT1	DNAH5	JAK2	PCSK5	SRRT
AKT2	DNAH9	JAK3	PCYT1A	SSBP3
AKT3	DNAJC11	KCNAB2	PDCD6	SSH2
ALDH1A3	DNAJC9	KCNH6	PDE1C	SSPO
ALDH2	DNMT3A	KCNQ2	PDE2A	ST18
ALG5	DNTTIP1	KDM4A	PDE4DIP	ST6GALNAC1
ALK	DOCK11	KDM6A	PDGFRA	STAG2

ALX4	DOCK3	KDR	PDGFRB	STAT1
AMOT	DOT1L	KEAP1	PDIA5	STAT3
ANK2	DPP10	KIAA0195	PDILT	STAT4
ANKRD13D	DPP4	KIAA0226	PDK1	STAT6
ANKRD20A4	DRGX	KIAA0319	PDRG1	STK11
ANKRD27	DUOX1	KIAA0922	PEX6	STK11IP
ANKRD28	DYSF	KIAA1191	PGAP1	STK31
ANKRD30A	DZANK1	KIAA1199	PHACTR3	STX3
ANKRD30B	ECHDC1	KIAA1211L	PHF20L1	SULT1A4
ANKRD36B	EDN1	KIF13A	PHYH	SUPT5H
ANO2	EEF1A1	KIF1B	PI4KB	SUPT6H
AP1B1	EFCAB5	KIF26B	PIK3CA	SYCP2L
AP1G2	EFCAB6	KIF5B	PIK3CB	SYK
AP3B1	EFCAB7	KIFAP3	PIK3R1	SYNE1
APAF1	EFHA2	KIFC1	PIK3R2	SYNE2
APC	EFNA5	KIR2DL3	PIP4K2C	SYNJ2
APLP2	EGFR	KIR3DL3	PIP5K1C	TAF1B
APMAP	EIF1AX	KIT	PIWIL1	TAF6
APPL2	EIF2B5	KLHL1	PKD1L2	TARBP1
AQP12A	EIF2C2	KLHL14	PKHD1	TBC1D1
AR	EIF3E	KLK1	PKLR	TBC1D21
ARAF	EIF3I	KMT2B	PLAC8	TBC1D3
ARFGAP1	EIF4ENIF1	KMT2C	PLCB4	TBC1D5
ARFRP1	EIF4H	KRAS	PLCZ1	TBL1X
ARHGAP35	ELAVL3	KRT2	PLEC	TBP
ARHGAP40	ELL3	KRT9	PLK2	TBX15
ARHGEF1	EMID2	KRTAP5-5	PLOD3	TBX22
ARHGEF7	ENPP2	KTN1	PLXNA1	TBX3
ARNTL	ENTPD6	L3MBTL1	PMS1	TCF20
ARPC4-TTLL3	EPB41L2	LARP1	PMS2	TCF4
ASH2L	EPB41L4B	LCN10	POLDIP2	TCP10
ASTN1	EPHA2	LCT	POLE	TCP11
ASXL2	EPHA3	LCTL	POLR2J	TEK
ATAD2B	EPHA5	LETM1	POLR3B	TERT
ATG9B	EPHB1	LGALS13	POLR3GL	TESC
ATM	EPS8L3	LILRB3	POLRMT	TEX35
ATP10B	ERBB2	LILRB4	POM121L12	TFDP1
ATP10D	ERBB3	LIPN	POTEG	TGDS
ATP12A	ERBB4	LMAN1L	PPA1	TGM2

ATP2C1	ERCC1	LMBR1L	PPDPF	TGM5
ATP6V0A2	ERG	LPCAT4	PPEF1	THBS2
ATP8B2	ESD	LPHN3	PPFIBP2	THEM5
ATR	ESR1	LRBA	PPIL2	THOC1
ATXN2	ETNK2	LRP1B	PPP1R17	THSD7A
ATXN7L2	ETV6	LRP2	PPP4R4	THSD7B
AURKA	EXOC4	LRP4	PQBP1	TIMD4
AURKB	EXOC5	LRRC16B	PREB	TIMM44
AXL	EXOC6	LRRC2	PREX2	TIMP3
BAP1	EXOC7	LRRC7	PRKAA1	TJP3
BAX	EXTL3	LRRC72	PRKACA	TLE1
BBS9	EYA4	LRRD1	PRKAG3	TLL1
BCAS1	EZH2	LRRFIP2	PRKCD	TMC2
BCAS2	F8	LRSAM1	PRKDC	TMED8
BCL2	F9	LTBP1	PRKX	TMEM104
BCL2L11	FAH	LUC7L2	PRRX1	TMEM120B
BCR	FAM114A2	LUZP4	PRSS1	TMEM132D
BLOC1S1	FAM131B	MAEL	PRUNE	TMEM145
BMPR1B	FAM135B	MAGI1	PSG2	TMEM247
BRAF	FAM13C	MAN2A1	PSG5	TMEM80
BRCA1	FAM157B	MAP2	PSIP1	TMEM87A
BRCA2	FAM177B	MAP2K1	PSMB1	TMPRSS2
BRD2	FAM21A	MAP2K2	PSMB5	TMTC4
BRD3	FAM3A	MAP2K4	PSMC4	TMX3
BRD4	FAM49A	MAP3K1	PSMC6	TNFAIP6
BRF1	FAM49B	MAP4K1	PSTPIP1	TNFSF4
BRSK2	FAM5C	MAPK1	PTBP3	TNN
BRWD3	FAM86B1	MAPK3	PTCD3	TNNT1
BSG	FAN1	MAPKAPK3	PTCH1	TNR
BTK	FANCC	MAPRE3	PTCH2	TNS3
BTNL3	FASTK	MAST1	PTEN	TOP1
BTRC	FAT1	MBIP	PTGES3L-AARSD1	TP53
C11orf30	FATE1	MBTPS2	PTGS2	TP53BP1
C12orf5	FBN2	MCF2L2	PTPLAD1	TPCN1
C19orf38	FBXW7	MCL1	PTPN11	TPH2
C1orf112	FCGR2A	MCOLN2	PTPN13	TPMT
C1orf35	FCGR2B	MDGA2	PTPRA	TPTE
C1QA	FCGR3A	MDM2	PTPRD	TRIM33

C1S	FDCSP	MDM4	PTPRM	TRIM51
C20orf112	FGFR1	MDN1	PYHIN1	TRIM58
C2orf47	FGFR2	MED12	QRICH2	TRIML1
C2orf62	FGFR3	MED23	RAB1B	TRIO
C7orf53	FGFR4	MEFV	RAB3GAP2	TRIP11
C9orf114	FLCN	MET	RAB6A	TRMT112
C9orf43	FLNC	METTL14	RAC2	TRPC5
CACNA1A	FLOT2	METTL5	RAF1	TRUB1
CACNA1D	FLT1	MGAM	RALBP1	TSC1
CACNA1E	FLT3	MICALL1	RAPGEF2	TSC2
CADM2	FLT3LG	MID1	RARA	TSGA10
CAMKK1	FLT4	MIER2	RARB	TSKS
CAPRIN1	FMN2	MITF	RASEF	TSPAN12
CARS	FMNL3	MLH1	RB1	TSR2
CARS2	FNDC4	MLH3	RBM6	TTF2
CASC4	FNIP2	MLL3	RBMX	TTN
CASP8	FOLH1	MLPH	RCC1	TUBA3C
CASP8AP2	FOXA1	MORC1	REC8	TUBGCP4
CASQ2	FOXJ2	MORN1	REG1B	TUBGCP5
CATSPER2	FOXL2	MPL	RELN	TYK2
CBFB	FRG1	MRPL1	RERE	TYRP1
CBL	FRG2B	MRPL24	RET	U2AF1
CBX4	FRMD4A	MRPS18B	RFWD2	U2AF2
CCDC155	FRMPD2	MS4A1	RFX3	UBASH3A
CCDC159	FRMPD4	MSH2	RHEB	UBE2Q1
CCDC17	FSD2	MSH3	RHOA	UBE4B
CCND1	FSHR	MSH6	RICTOR	UCHL3
CCND2	FUBP1	MSI1	RNF215	UCK2
CCND3	FUNDC1	MTA2	RNF219	UGT8
CCNE1	GAB2	MTM1	RNF43	ULK3
CCT3	GAB3	MTOR	ROCK1	UMOD
CCT6B	GABRD	MTR	ROS1	UNC13A
CD1E	GAD2	MTTP	RPL22	UNC13D
CD274	GALNT13	MUC5B	RPL36A	UNC5D
CD300LF	GALNT14	MUS81	RPS5	USP12
CD5L	GATA3	MYB	RPS6KA1	USP34
CD9	GFRAL	MYBPC2	RPS6KB1	USP39
CD97	GIGYF1	MYC	RPTOR	USP45
CD99	GINS4	MYCBP2	RPUSD4	USP48

CDH1	GIPR	MYD88	RREB1	VAV1
CDH18	GKN2	MYH15	RRP7A	VEGFA
CDH24	GLB1L3	MYH2	RUNDC3A	VEZF1
CDH26	GLYR1	MYH4	RUNX1	VHL
CDK11A	GMDS	MYH8	RYR2	VILL
CDK12	GNA11	MYH9	RYR3	VIT
CDK13	GNAQ	MYL5	SAFB2	VPS13A
CDK14	GNAS	MYL6	SAG	VPS33B
CDK18	GNPTAB	MYLK2	SAGE1	VSIG4
CDK19	GOLGA4	MYO3A	SAMD8	WAS
CDK4	GPAT2	MYOM1	SCN10A	WASL
CDK6	GPATCH2	NACAD	SCN3A	WDR44
CDK8	GPR114	NARF	SCN7A	WDR52
CDKN1A	GPR125	NAT10	SCN9A	WDR62
CDKN1B	GPR133	NAV3	SDK2	WDR66
CDKN2A	GPR144	NBPF1	SEC14L4	WDR72
CDKN2B	GPS2	NBPF10	SEC24B	WDTC1
CDS1	GRIA3	NCF2	SEH1L	WLS
CEACAM20	GRIK2	NCKAP1	SELP	WSCD2
CECR2	GUCY1A3	NCOR1	SEMA6A	WWP2
CELA2B	GUCY2C	NCOR2	SEPT12.	XBP1
CGN	GYLTL1B	NEK5	SERPINA7	XPO1
CHD3	HAAO	NELL1	SETD1B	XPO4
CHD4	HAP1	NF1	SETD2	XPO5
CHD6	HAUS5	NF2	SF1	XRCC1
CHEK1	HAUS6	NFE2L2	SF3B1	ZAP70
CHEK2	HCN1	NIPBL	SF3B14	ZBTB8OS
CHI3L1	HDAC1	NLGN3	SF3B3	ZC3H13
CISD3	HDAC4	NLRC3	SGCZ	ZC3H7B
CLCN7	HDAC6	NLRP4	SGIP1	ZDHHC11
CLEC16A	HEATR7B2	NMI	SGK1	ZFC3H1
CLINT1	HECTD4	NOP2	SGPL1	ZFR
CNGB3	HECW1	NOS1	SH2D3A	ZMYM4
CNKSR2	HECW2	NOS2	SH3BGR	ZNF143
CNOT3	HGF	NOTCH1	SH3PXD2A	ZNF350
CNOT4	HID1	NOTCH2	SHISA4	ZNF385A
CNTN1	HIST1H3B	NOTCH3	SI	ZNF414
CNTN4	HLA-DRB1	NOTCH4	SIDT2	ZNF512B
CNTN5	HLA-DRB5	NRAS	SIK3	ZNF541

CNTNAP3B	HMCN1	NRXN1	SIM1	ZNF563
CNTNAP5	HMHA1	NRXN2	SIM2	ZNF614
COASY	HNF4A	NT5C3L	SLC13A3	ZNF687
COL14A1	HOMER2	NTM	SLC17A6	ZNF705B
COL16A1	HPS3	NTRK1	SLC17A8	ZNF705G
COL19A1	HPS4	NTRK3	SLC25A1	ZNF711
COL1A1	HRAS	NUDCD2	SLC25A30	ZNF804B
COL25A1	HSPA12B	NUP205	SLC26A3	ZSWIM8
COL4A5				
COL4A6				
COL5A1				
COL5A2				
COL5A3				
COL6A5				
COL6A6				
COL9A1				
COPA				
COPG1				
CPA1				
CPSF3				
CPSF6				
CRKL				
CRTAM				
CRTAP				
CRYBG3				
CSF1R				
CSMD1				
CSMD3				
CSN3				

Supplementary Table S2. Somatic point mutations identified in 100 plasma samples					
Patient ID	Gene Symbol	cHGVS	pHGVS_ad	Mutation AF (%)	Subtype (1=HR+/HER2, 2=HR+/HER2+, 3=HR-/HER2+, 4=HR-/HER2-)
1	TP53	c.[503A>G]	p.[H168R]	2.87	3
1	FLT4	c.[2180C>T]	p.[A727V]	1.10	3
1	CARD11	c.[1656_1657insC]	p.[P553fs*38]	1.33	3
1	EZH2	c.[1544A>G]	p.[K515R]	1.11	3
1	FPGS	c.[416G>T]	p.[R139L]	2.14	3
1	CHEK1	c.[122A>G]	p.[D41G]	2.22	3
1	ASPSCR1	c.[951_965delCGTGGACCGGGAGCC]	p.[D319_V323del]	2.14	3
1	STK11	c.[883G>A]	p.[A295T]	1.18	3
1	PRX	c.[1646C>T]	p.[P549L]	1.13	3
1	PAK3	c.[546A>T]	p.[E182D]	1.86	3
1	ROS1	c.[2713A>G]	p.[T905A]	2.90	3
1	ARID1B	c.[1379_1381delCGG]	p.[A460del]	2.72	3
1	PTCH1	c.[38_40delGCG]	p.[G17del]	2.68	3
1	NKX2-1	c.[711_713delGGG]	p.[G241del]	27.09	3
1	CEBPA	c.[589_590insACCCGC]	p.[H195_P196dup]	38.67	3
2	PIK3CA	c.[3140A>G]	p.[H1047R]	50.50	1
2	TP53	c.[578A>T]	p.[H193L]	37.24	1
2	PTEN	c.[801+1G>C]	-	2.46	1
2	SF3B1	c.[2098A>G]	p.[K700E]	1.09	1
2	PRKCB	c.[950C>A]	p.[P317Q]	43.09	1
2	ROS1	c.[5743G>A]	p.[G1915R]	1.17	1
2	MYC	c.[144G>C]	p.[Q48H]	1.07	1
2	GATA3	c.[925-3_925-2delCA]	-	2.24	1
2	KDM5C	c.[2087C>A]	p.[A696D]	1.14	1
3	BRCA2	c.[5635G>T]	p.[E1879*]	1.00	1
3	FGFR3	c.[770C>T]	p.[A257V]	1.03	1
3	CAMK2G	c.[287G>T]	p.[G96V]	1.22	1
3	TNFSF11	c.[185G>T]	p.[C62F]	2.17	1
3	NOTCH3	c.[1346G>A]	p.[R449H]	1.21	1
3	FPGS	c.[1307C>T]	p.[A436V]	1.00	1

3	PTEN	c.[804C>A]	p.[D268E]	19.89	1
3	LRRK2	c.[1562G>T]	p.[R521M]	2.53	1
3	DOT1L	c.[3041G>T]	p.[G1014V]	13.10	1
3	CEBPA	c.[589_590insACCCGC]	p.[H195_P196dup]	6.33	1
3	AR	c.[194A>T]	p.[Q65L]	1.01	1
4	AR	c.[1369_1371delGGC]	p.[G457del]	13.10	4
4	KMT2C	c.[3340T>C]	p.[C1114R]	6.33	4
4	SF3B1	c.[2960C>A]	p.[P987H]	1.01	4
4	PDGFRB	c.[1811G>A]	p.[R604H]	1.29	4
4	KLF4	c.[779C>A]	p.[P260Q]	1.26	4
4	CAMK2G	c.[66-1G>T]	-	1.05	4
4	SUFU	c.[71delC]	p.[P24fs*72]	1.41	4
4	SMARCA4	c.[896C>A]	p.[P299H]	1.11	4
4	EPHB6	c.[493_495delCCC]	p.[P165del]	4.04	4
4	MYC	c.[144G>C]	p.[Q48H]	1.06	4
4	IRS2	c.[568G>A]	p.[A190T]	1.19	4
4	NF1	c.[2602G>T]	p.[G868C]	1.19	4
5	NF1	c.[2205T>A]	p.[Y735*]	3.59	1
5	MED12	c.[4642C>T]	p.[Q1548*]	1.90	1
5	TET2	c.[4546C>T]	p.[R1516*]	3.43	1
5	NTRK1	c.[1954G>T]	p.[A652S]	49.06	1
5	WT1	c.[394_396delCCG]	p.[P132del]	1.05	1
5	SUZ12	c.[161_163delCCT]	p.[S59del]	21.82	1
5	CEBPA	c.[589_590insACCCGC]	p.[H195_P196dup]	2.40	1
5	NTRK1	c.1954G>T	p.A652S	47.70	1
5	ALK	c.950G>T	p.R317I	1.50	1
5	ESR1	c.1613A>G	p.D538G	46.00	1
5	EGFR	c.3286T>A	p.S1096T	0.30	1
5	MED12	c.5285A>G	p.K1762R	0.60	1
6	TOP1	c.[2245C>T]	p.[R749W]	4.30	2
6	CEBPA	c.[589_590insACCCGC]	p.[H195_P196dup]	1.64	2
7	CDKN2A	c.42C>A	p.D14E	0.50	3
7	FBXW7	c.608C>A	p.S203*	45.40	3
7	MTOR	c.4918C>T	p.R1640W	13.30	3
7	NF2	c.79G>A	p.V27I	8.10	3
7	STK11	c.40G>T	p.E14*	4.20	3
7	TP53	c.702C>A	p.Y234*	80.00	3
7	BRCA2	c.4957A>T	p.T1653S	40.00	3
8	TP53	c.734G>T	p.G245V	0.30	2

8	AXL	c.1280G>A	p.R427H	0.40	2
8	ESR1	c.1613A>G	p.D538G	0.40	2
8	BCOR	c.1423T>G	p.L475V	0.50	2
8	CHD2	c.493A>Tp	p.S165C	0.50	2
8	PRPF4OB	c.2554C>T	p.R852C	0.80	2
8	KIT	c.356T>G	p.L119R	0.60	2
8	IDH1	c.481G>A	p.G161R	0.70	2
8	LYN	c.362T>G	p.L121R	3.20	2
8	SRC	c.1289A>G	p.K430R	2.40	2
8	EZH2	c.1544A>G	p.K515R	1.20	2
8	PIK3CA	c.3139C>T	p.H1047Y	1.10	2
9	AKT3	c.899C>T	p.A300V	0.80	2
9	CTNNB1	c.95A>T	p.D32V	1.00	2
9	PIK3CA	c.2087G>T	p.G696V	1.10	2
9	PIK3R1	c.2135T>A	p.V712D	1.20	2
9	ESR1	c.893T>A	p.I298N	0.50	2
9	ESR1	c.1679T>A	p.V560E	0.60	2
9	MET	c.54G>T	p.L18F	0.60	2
9	TSC1	c.1930T>C	p.S644P	13.80	2
9	PTPN11	c.1162G>A	p.V388I	0.70	2
9	FLT3	c.1927G>A	p.V643I	0.50	2
9	TSC2	c.4294G>A	p.A1432T	0.30	2
10	BRCA2	c.1744A>C	p.T582P	1.00	3
10	CHD1	c.2530A>G	p.S844G	0.80	3
11	TP53	c.524G>A	p.R175H	0.90	1
11	AR	c.173A>T	p.Q58L	1.60	1
12	ALK	c.3467G>T	p.C1156F	2.10	1
12	FBXW7	c.620C>A	p.T207K	16.00	1
12	ROS1	c.3488A>T	p.K1163M	0.40	1
12	CDKN1B	c.169C>T	p.Q57*	0.40	1
12	AKT1	c.49G>A	p.E17K	21.80	1
12	TP53	c.751A>T	p.I251F	17.70	1
13	MDM4	c.460G>A	p.D154N	2.10	1
13	ATR	c.859C>A	p.Q287K	1.30	1
13	KDR	c.1111C>A	p.L371I	0.50	1
13	CDKN2AA	c.305C>A	p.A102E	0.50	1
13	PTCH1	c.367A>C	p.T123P	0.40	1
13	FLT1	c.758C>A	p.T253N	0.60	1
13	BRCA1	c.2576A>T	p.N859I	0.40	1

13	JAK3	c.244T>G	p.F82V	0.90	1
13	CHEK2	c.1367C>G	p.S456*	0.60	1
14	CTNNA1	c.1619G>A	p.R540H	0.30	3
14	TSC2	c.1444G>A	p.E482K	0.80	3
14	TP53	c.659A>G	p.Y220C	0.40	3
14	AR	c.173A>T	p.Q58L	1.30	3
15	PIK3CA	c.2309G>A	p.R770Q	1.10	2
15	PIK3CA	c.3141T>G	p.H1047Q	4.10	2
15	NOTCH4	c.3697T>A	p.C1233S	3.00	2
15	SYK	c.1091A>G	p.K364R	0.40	2
15	TP53	c.351delG	p.T118Qfs*5	0.40	2
16	ERBB2	c.2329G>T	p.V777L	0.80	3
16	TP53	c.993+2T>G	.	0.50	3
16	MET	c.3562C>T	p.R1188*	1.30	3
16	CCND1	c.641G>T	p.G214V	2.20	3
16	TP53	c.574C>T	p.Q192*	0.50	3
16	SYK	c.1091A>G	p.K364R	4.50	3
17	MTOR	c.5577C>G	p.S1859R	0.40	3
17	EPHA2	c.1987G>A	p.E663K	0.70	3
17	ABL2	c.103G>A	p.D35N	4.60	3
17	EZH2	c.1544A>G	p.K515R	3.50	3
17	ABL1	c.1425T>A	p.C475*	0.60	3
17	NOTCH1	c.7531A>C	p.T2511P	0.40	3
17	CDK2	c.225A>T	p.K75N	0.60	3
18	PIK3CA	c.1633G>A	p.E545K	0.30	4
18	TSC2	c.5197A>C	p.T1733P	0.30	4
19	ERBB4	c.1981C>A	p.L661I	0.50	1
19	HGF	c.118C>G	p.H40D	0.40	1
19	NF1	c.7798G>C	p.E2600Q	15.70	1
19	NOTCH3	c.2884G>T	p.E962*	0.50	1
20	BRCA2	c.8187G>T		0.90	4
20	RAD51D	c.481-8C>T		0.60	4
21	IKBKE	c.380G>T	p.R127L	0.40	2
21	MLL	c.8111G>T	p.R2704L	0.40	2
21	MLL2	c.58C>A	p.P20T	0.40	2
21	C1S	c.1486C>T	p.R496W	0.40	2
21	BCORL1	c.4391G>T	p.C1464F	0.30	2
21	CBL	c.1549G>T	p.G517*	27.30	2
21	MLL3	c.12433C>A	p.R4145S	0.40	2

21	PAK3	c.110T>G	p.M37R	3.20	2
21	XPO1	c.656C>A	p.A219D	1.20	2
22	CEBPA	c.590_591insACCCGC	p.P198_A199dup	0.20	3
22	ERBB4	c.743C>A	p.A248D	0.40	3
22	ATM	c.278A>G	p.K93R	1.80	3
22	FGF14	c.419C>A	p.P140Q	0.40	3
22	MLL3	c.6668C>A	p.A2223E	0.20	3
22	MSH2	c.2204T>A	p.I735N	0.20	3
22	NCOR1	c.4834A>G	p.T1612A	0.10	3
22	TET2	c.5090G>T	p.G1697V	6.40	3
23	TP53	c.375+2T>G	.	3.40	3
23	CD79B	c.278A>G	p.K93R	1.40	3
23	EML4	c.2571G>T	p.M857I	0.70	3
23	FLT1	c.1573G>A	p.A525T	7.70	3
24	AR	c.1369_1371delGGC	p.G457del	5.90	2
24	IRS2	c.228C>A	p.Y76*	6.10	2
24	MAPK3	c.341C>A	p.A114D	7.40	2
24	DOT1L	c.1123G>T	p.G375C	12.20	2
24	EGFR	c.2899G>T	p.E967*	0.50	2
24	EML4	c.694C>T	p.R232C	1.10	2
24	EP300	c.3200G>T	p.R1067L	0.90	2
24	PIK3C2B	c.1803C>A	p.C601*	0.70	2
24	PIK3CA	c.3140A>G	p.H1047R	1.00	2
24	PRKAA1	c.808C>A	p.L270I	8.40	2
24	PTCH1	c.2760C>A	p.Y920*	7.30	2
24	SUFU	c.562G>A	p.V188M	3.80	2
24	TOP2B	c.3182C>A	p.S1061Y	2.20	2
24	U2AF1	c.196C>T	p.R66C	1.30	2
25	CCND3	c.311C>T	p.A104V	0.50	2
25	EPPK1	c.6716G>A	p.R2239H	0.50	2
25	ERBB2	c.3235G>A	p.E1079K	0.40	2
25	FCGR2A	c.245C>T	p.P82L	20.30	2
25	FCGR3A	c.302G>A	p.S101N	17.40	2
25	PIK3CA	c.3140A>G	p.H1047R	14.50	2
26	ATR	c.3581G>A	p.R1194K	7.20	2
26	BCL6	c.119G>A	p.R40H	4.20	2
26	FGFR4	c.770C>T	p.A257V	3.30	2
26	JAK1	c.619C>T	p.Q207*	1.50	2
26	MLL2	c.15137C>A	p.P5046H	0.80	2

26	ROBO2	c.1457A>G	p.Y486C	0.40	2
27	AR	c.[1369_1380delGGCGGC GGCGGC]	p.G470_G473del	0.30	3
27	ARHGAP35	c.242G>A	p.R81H	0.30	3
27	AXIN2	c.205G>A	p.A69T	0.30	3
27	CDK12	c.610A>G	p.R204G	45.40	3
27	IKBKE	c.1822G>T	p.G608C	36.20	3
27	MAX	c.299G>A	p.R100H	30.00	3
27	NF2	c.1207G>A	p.A403T	17.60	3
27	NOTCH4	c.4307C>T	p.A1436V	13.10	3
27	SOX17	c.364G>T	p.E122*	11.70	3
27	TP53	c.497C>G	p.S166*	10.90	3
27	TRRAP	c.7309A>G	p.R2437G	8.80	3
28	TP53	c.818G>A	p.R273H	3.50	3
28	ROS1	c.6316G>A	p.A2106T	3.20	3
28	PML	c.851G>C	p.R284P	3.10	3
28	IRF4	c.31G>T	p.E11*	2.90	3
28	MLL4	c.6559G>A	p.A2187T	2.60	3
28	B4GALT3	c.926G>A	p.R309H	2.50	3
28	NPM1	c.545A>G	p.D182G	2.20	3
28	SETD2	c.22C>T	p.P8S	2.20	3
28	VEGFA	c.826C>T	p.P276S	2.10	3
28	ZNF217	c.77G>A	p.S26N	1.90	3
29	TP53	c.392A>G	p.N131S	1.50	2
29	EPPK1	c.6716G>A	p.R2239H	1.40	2
29	ATM	c.5788G>C	p.D1930H	1.10	2
29	ESR1	c.1610A>C	p.Y537S	0.90	2
29	FCGR3A	c.302G>A	p.S101N	0.80	2
29	MET	c.890A>G	p.E297G	0.70	2
29	NFE2L3	c.1244C>G	p.P415R	0.70	2
29	ROBO1	c.965C>A	p.A322D	0.60	2
29	SMAD3	c.261C>G	p.I87M	0.50	2
29	USP9X	c.4507G>C	p.E1503Q	0.70	2
29	DOCK2	c.4046G>T	p.G1349V	0.70	2
29	SRSF1	c.401G>A	p.W134*	0.40	2
29	STAT5B	c.1976G>A	p.R659H	22.30	2
29	RAC1	c.502G>T	p.V168L	22.10	2
29	WHSC1L1	c.1893C>A	p.F631L	1.50	2
29	SMARCA4	c.3599G>A	p.R1200H	0.60	2

29	C1S	c.371C>T	p.A124V	0.30	2
29	EGFR	c.3038C>T	p.A1013V	0.30	2
29	FLI1	c.433G>A	p.A145T	0.30	2
29	TRRAP	c.973G>A	p.A325T	0.30	2
30	FGFR3	c.1138G>A	p.G380R	0.30	3
30	FGFR3	c.1103A>G	p.E368G	0.20	3
30	KIF5B	c.2792C>T	p.A931V	1.18	3
30	PIK3R2	c.1217G>A	p.R406H	3.62	3
30	PRPF40B	c.1945C>T	p.R649W	2.33	3
30	ARID1B	c.2342C>T	p.A781V	1.93	3
30	ARID2	c.4773+1G>T	.	1.79	3
30	ERCC3	c.698C>A	p.S233Y	1.63	3
30	PIK3C2B	c.2558T>C	p.L853P	1.55	3
30	EP300	c.6603A>G	p.I2201M	1.55	3
31	BRAF	c.964G>A	p.A322T	1.26	2
31	MLL	c.4778G>A	p.R1593H	1.13	2
31	RNASEL	c.505G>T	p.A169S	1.44	2
31	ZNF703	c.1454C>T	p.T485M	1.15	2
31	CREBBP	c.6761T>C	p.L2254P	1.32	2
31	NR3C1	c.218C>T	p.A73V	1.08	2
31	GATA2	c.572C>T	p.A191V	1.27	2
31	MLL	c.11455C>T	p.R3819C	1.04	2
31	VHL	c.430G>A	p.G144R	1.25	2
31	EP300	c.5705C>T	p.A1902V	1.23	2
31	MLL3	c.4672C>T	p.R1558W	1.05	2
32	TP53	c.706T>A	p.Y236N	4.53	3
32	ROBO1	c.4178C>A	p.S1393*	3.02	3
32	NOTCH4	c.2288A>T	p.Q763L	2.12	3
32	PRKCA	c.877G>A	p.G293R	2.03	3
32	ASPSCR1	c.791C>T	p.S264L	2.17	3
32	C1R	c.1483C>T	p.R495C	1.20	3
32	CHD4	c.25T>C	p.S9P	1.28	3
32	KAT6A	c.3390A>C	p.K1130N	1.93	3
32	CDK12	c.3724C>T	p.R1242*	1.95	3
32	FGF7	c.363G>T	p.M121I	1.07	3
32	PRX	c.3890A>G	p.E1297G	1.43	3
32	SPEN	c.1595A>G	p.Y532C	1.35	3
33	PIK3CA	c.3140A>G	p.H1047R	29.92	2
33	TOP2A	c.1627-1G>C	.	26.38	2

33	TP53	c.659A>G	p.Y220C	21.51	2
33	NCOA1	c.709G>A	p.E237K	7.65	2
33	PIK3R1	c.889G>A	p.E297K	7.61	2
33	ATM	c.5318A>G	p.K1773R	1.05	2
33	CREBBP	c.5713C>A	p.P1905T	1.70	2
33	DAXX	c.1112G>A	p.R371Q	1.53	2
33	DNMT1	c.2309C>T	p.A770V	1.17	2
33	ELAC2	c.2131G>A	p.V711M	2.43	2
33	ELAC2	c.1583A>T	p.Y528F	1.50	2
33	ELMO1	c.1115C>T	p.T372M	1.09	2
33	FANCA	c.1511G>A	p.R504H	1.01	2
33	FAT3	c.881C>T	p.A294V	1.24	2
33	HDAC4	c.230C>T	p.A77V	1.36	2
33	HDAC6	c.1694A>G	p.N565S	1.10	2
33	IKBKE	c.1789G>A	p.V597M	1.38	2
33	MAPK8	c.205C>T	p.R69W	1.44	2
33	MED12	c.6226C>T	p.Q2076*	1.35	2
33	MLL	c.4778G>A	p.R1593H	1.02	2
33	NCOA2	c.3868C>T	p.R1290W	2.04	2
33	NF1	c.8138G>T	p.R2713L	2.54	2
33	NR3C1	c.1477A>T	p.T493S	1.02	2
33	PARP1	c.718G>T	p.A240S	1.08	2
33	PHF6	c.44G>A	p.R15H	1.22	2
33	PRKDC	c.11479G>A	p.A3827T	1.05	2
33	PTCH2	c.2211C>G	p.Y737*	1.17	2
33	RNF43	c.1166G>A	p.R389H	1.25	2
33	SPEN	c.7796C>T	p.S2599L	1.20	2
33	TOP1	c.1496C>T	p.A499V	1.04	2
33	TSHZ3	c.2293A>G	p.T765A	1.67	2
33	USP9X	c.3994G>A	p.A1332T	1.20	2
34	PIK3CA	c.1035T>A	p.N345K	35.81	2
34	PALB2	c.2534_2535insA	p.A846Sfs*3	2.34	2
34	FOXP1	c.1183G>A	p.A395T	1.08	2
34	KDM5A	c.1010G>C	p.C337S	2.37	2
34	PRPF40B	c.1839_1846delGGCTGCCG	p.R613Sfs*13	1.39	2
34	RET	c.169C>T	p.R57W	1.01	2
35	TSHZ3	c.2743C>T	p.R915W	17.23	2
35	RARB	c.1078C>T	p.R360*	12.80	2

35	NCOA2	c.1516C>T	p.Q506*	10.44	2
35	ACVR2A	c.949G>A	p.A317T	1.34	2
35	ASXL1	c.3503G>C	p.S1168T	1.34	2
35	CYP17A1	c.1046G>A	p.R349H	1.11	2
35	DOCK2	c.3568G>A	p.G1190S	2.89	2
35	EP300	c.3841C>T	p.R1281*	2.21	2
35	EPHA5	c.1951A>C	p.I651L	1.49	2
35	FANCA	c.3418A>T	p.N1140Y	1.02	2
35	FGFR3	c.1138G>A	p.G380R	1.53	2
35	FLT1	c.1573G>A	p.A525T	1.08	2
35	GRIN2A	c.1310G>A	p.R437Q	2.06	2
35	IGF1R	c.3482A>G	p.Y1161C	2.16	2
35	IRF4	c.275C>A	p.T92N	1.10	2
35	KDM5A	c.2797G>A	p.A933T	1.02	2
35	MDM2	c.212G>A	p.R71Q	2.54	2
35	MSH5	c.1292G>A	p.R431H	1.34	2
35	NAV3	c.5696C>G	p.S1899C	2.34	2
35	NOTCH4	c.419G>A	p.R140H	1.56	2
35	PAK3	c.546A>T	p.E182D	1.17	2
35	PDGFRA	c.2936G>A	p.R979H	2.34	2
35	PMS1	c.1162A>C	p.I388L	1.35	2
35	PSMB1	c.494C>A	p.A165D	1.20	2
36	ATRX	c.737G>A	p.R246H	3.62	2
36	CASP8	c.683C>T	p.S228F	3.19	2
36	CD33	c.743C>G	p.S248*	3.08	2
36	CHD2	c.2343G>T	p.E781D	2.83	2
36	SETBP1	c.3271C>T	p.P1091S	1.67	2
36	TGFBR2	c.1610G>A	p.R537H	2.13	2
36	PIK3C2G	c.3740G>A	p.S1247N	1.71	2
37	DNMT1	c.2621A>G	p.D874G	1.48	2
37	EIF4A2	c.716A>G	p.K239R	1.72	2
37	NFE2L2	c.371C>T	p.A124V	1.24	2
37	TAF1	c.3650G>A	p.R1217H	1.22	2
37	TOP2A	c.3595A>G	p.K1199E	1.42	2
37	ZRSR2	c.1355G>A	p.R452H	1.11	2
37	MTOR	c.4286C>T	p.A1429V	1.15	2
37	MYC	c.144G>C	p.Q48H	1.01	2
37	ALK	c.224C>A	p.P75Q	1.04	2
37	ZNF217	c.1073C>T	p.A358V	1.10	2

37	ELAC2	c.397C>A	p.L133I	1.03	2
38	PIK3CA	c.1035T>A	p.N345K	13.80	2
38	ELF3	c.281G>A	p.R94Q	11.76	2
38	SPEN	c.2137C>T	p.R713W	12.67	2
38	ERBB4	c.2935C>G	p.R979G	11.69	2
38	PIK3CB	c.2948G>A	p.R983H	12.32	2
38	MTOR	c.6965G>A	p.R2322H	11.04	2
38	NOTCH2	c.272G>A	p.R91Q	13.57	2
38	SETBP1	c.3636C>A	p.H1212Q	10.90	2
38	TSPAN3	c.608A>G	p.K203R	5.45	2
38	MC1R	c.667C>T	p.R223W	9.71	2
38	ATR	c.2921C>T	p.T974M	9.47	2
38	GAB2	c.1424T>C	p.V475A	6.94	2
38	C11orf30	c.3236C>T	p.S1079L	6.38	2
38	KAT6A	c.4139A>T	p.H1380L	3.99	2
38	RAD51C	c.209C>T	p.T70I	9.12	2
38	TSHR	c.2272G>A	p.E758K	5.86	2
38	ASXL1	c.4099G>A	p.V1367I	5.68	2
38	KDR	c.1040G>A	p.R347H	5.54	2
38	TP53	c.733G>A	p.G245S	4.98	2
38	SMO	c.611A>G	p.K204R	4.66	2
38	ATM	c.8842A>G	p.I2948V	3.51	2
38	PRKDC	c.4819G>A	p.E1607K	3.25	2
38	BCORL1	c.3740A>G	p.Q1247R	2.65	2
38	TP53	c.376-1G>A	.	2.39	2
38	CIC	c.798T>A	p.D266E	2.25	2
38	CHD2	c.5335C>G	p.P1779A	2.70	2
38	ERBB2	c.677G>A	p.R226H	1.14	2
38	FGFR3	c.1138G>A	p.G380R	1.83	2
38	HSD17B3	c.839T>C	p.L280P	3.15	2
38	FH	c.97G>A	p.V33M	1.70	2
38	ALK	c.386G>T	p.G129V	1.69	2
38	JAK3	c.1208G>A	p.R403H	1.13	2
38	PIK3CA	c.1624G>A	p.E542K	1.12	2
39	NSD1	c.1538A>G	p.K513R	0.30	1
39	MLL3	c.11609A>G	p.K3870R	0.30	1
40	DNMT3A	c.1988C>G	p.S663W	0.30	2
40	CBL	c.1247G>C	p.C416S	0.30	2
40	ERBB2	c.1900T>C	p.C634R	0.40	2

41	PIK3CA	c.3140A>G	p.H1047R	1.50	1
41	FLT3	c.826C>A	p.H276N	1.10	1
41	TP53	c.817C>T	p.R273C	0.90	1
42	SYK	c.1091A>G	p.K364R	0.70	3
43	PIK3CA	c.1633G>A	p.E545K	27.90	1
43	ESR1	c.1613A>G	p.D538G	22.50	1
44	PIK3CA	c.3140A>T	p.H1047L	0.70	3
45	PIK3CA	c.1624G>A	p.E542K	0.60	3
45	TP53	c.332T>A	p.L111Q	0.50	3
45	EZH2	c.1529A>G	p.K510R	2.50	3
46	EZH2	c.1529A>G	p.K510R	0.60	1
46	PTCH1	c.443_445delGAG	p.G148del	0.60	1
46	ABL1	c.2029G>C	p.G677R	3.90	1
46	CCND1	c.839A>T	p.E280V	2.60	1
46	MDM2	c.272delT	p.L91Cfs*9	1.90	1
46	FLT1	c.1180G>A	p.D394N	1.50	1
46	GNAS	c.76C>T	p.R26C	1.00	1
47	ROBO3	c.1073C>T	p.A358V	0.60	2
48	MED12	c.5285A>G	p.K1762R	1.00	1
48	XRCC1	c.839G>A	p.R280H	0.50	1
48	JAK3	c.2470T>A	p.Y824N	0.40	1
48	NOTCH1	c.2108G>A	p.R703H	0.40	1
49	SHISA4	c.347G>A	p.R116H	0.50	3
49	TRIO	c.2116G>A	p.V706M	0.50	3
49	TSC1	c.1519T>C	p.F507L	0.60	3
49	FGFR2	c.820G>A	p.V274I	0.50	3
49	MLL4	c.2884C>T	p.R962C	28.40	3
50	MED12	c.1253G>A	p.R418H	20.60	2
50	RICTOR	c.1834C>T	p.L612F	8.20	2
50	DDR2	c.2512A>G	p.N838D	7.30	2
50	EPHA5	c.403C>T	p.R135W	6.70	2
50	IGF1R	c.2156G>A	p.R719H	2.40	2
50	CDKN2A	c.211C>T	p.R71C	1.20	2
50	ERBB2	c.1516C>T	p.R506W	1.10	2
50	TYRP1	c.1000G>A	p.A334T	0.90	2
50	TSC2	c.4852G>A	p.V1618I	0.50	2
51	GAB2	c.1139G>C	p.R380T	0.40	1
51	FOXA1	c.781C>G	p.R261G	0.40	1
51	SF3B1	c.2098A>G	p.K700E	0.40	1

52	MAP2K2	c.45C>G	p.N15K	0.40	3
52	TP53	c.747G>T	p.R249S	0.40	3
52	RET	c.79T>G	p.L27V	0.30	3
52	KRAS	c.183A>C	p.Q61H	25.40	3
52	DNMT3A	c.473T>C	p.L158P	61.30	3
52	EPHA3	c.1925T>G	p.L642R	0.40	3
52	MAP2K1	c.188T>G	p.L63R	0.90	3
53	DNMT3A	c.1618C>T	p.R540W	0.40	1
53	MED12	c.1730A>G	p.Q577R	0.40	1
54	MLL	c.2497A>T	p.T833S	0.40	3
55	TMPRSS	2c.589G>A	p.V197M	0.30	2
56	HCLS1	c.1274G>T	p.G425V	34.50	1
56	BRD3	c.719A>G	p.K240R	1.40	1
56	TP53	c.783T>A	p.S261R	0.50	1
57	ERBB4	c.3200G>A	p.R1067Q	1.30	1
57	PIK3CA	c.3140A>G	p.H1047R	1.30	1
57	TERT	c.-58-u232	6G>A-	1.30	1
57	MSH3	c.72G>C	p.L24F	1.10	1
57	PDGFRB	c.2980G>T	p.G994W	1.10	1
57	PDGFRB	c.1323G>T	p.M441I	1.00	1
57	NOTCH4	c.4801C>T	p.Q1601*	0.90	1
57	NOTCH4	c.388A>G	p.R130G	0.90	1
57	ESR1	c.1138G>C	p.E380Q	0.70	1
57	ESR1	c.1610A>C	p.Y537S	0.70	1
57	ESR1	c.1613A>G	p.D538G	0.60	1
57	ABL1	c.2489T>C	p.L830P	0.60	1
57	NOTCH1	c.337A>T	p.N113Y	0.50	1
57	MLH3	c.1252G>T	p.E418*	0.40	1
57	TP53	c.733G>A	p.G245S	0.40	1
58	ND			0.00	1
59	ATM	c.8311A>T	p.T2771S	25.00	1
60	TP53	c.722C>G	p.S241C	3.00	1
60	EPHA3	c.1234G>T	p.V412F	2.10	1
60	ESR1	c.1610A>C	p.Y537S	1.60	1
60	ESR1	c.1613A>G	p.D538G	1.40	1
60	ESR1	c.1609T>A	p.Y537N	1.20	1
60	P4HB	c.1160_1161insA	p.N387Kfs*23	0.60	1
60	RHOA	c.88T>G	p.F30V	0.50	1
61	MSH6	c.71C>T	p.S24L	25.00	1

61	PIK3CA	c.277C>T	p.R93W	2.90	1
61	PIK3CA	c.1633G>A	p.E545K	4.00	1
61	CDK13	c.872C>T	p.S291L	3.80	1
61	GNAS	c.842G>A	p.R281K	3.00	1
61	PTPN11	c.591T>G	p.Y197*	2.10	1
61	ESR1	c.1610A>C	p.Y537S	1.60	1
61	HGFc	.1940G>A	p.R647Q	1.40	1
61	IGF1R	c.2842G>A	p.V948M	1.20	1
61	HCLS1	c.580T>A	p.F194I	0.60	1
61	SRCc	.836G>A	p.G279D	0.50	1
62	AURKA	c.454G>A	p.E152K	4.80	1
62	FBXW7	c.2060G>T	p.G687V	0.60	1
62	MED12	c.220A>T	p.S74C	0.40	1
62	MTOR	c.4279G>A	p.E1427K	0.40	1
62	FGFR2	c.512C>T	p.A171V	0.40	1
62	NF2c	.1512C>A	p.S504R	0.40	1
62	ANO2	c.1396_1397GA>AT	p.D466I	0.40	1
62	NOTCH3	c.3143-2A>T	.	0.30	1
63	PIK3CA	c.1072A>T	p.T358S	0.30	2
63	PIK3CA	c.3140A>G	p.H1047R	27.30	2
63	ESR1	c.1609T>A	p.Y537N	0.40	2
63	ESR1	c.1610A>C	p.Y537S	3.20	2
63	ESR1	c.1613A>G	p.D538G	1.20	2
63	CCND1	c.287A>G	p.K96R	0.20	2
63	ERBB3	c.2615A>T	p.K872M	0.40	2
63	BRCA2	c.3459G>C	p.K1153N	1.80	2
63	RB1	c.2077G>T	p.E693*	0.40	2
63	AKT1	c.581A>C	p.H194P	0.20	2
63	ERBB2	c.2726A>G	p.E909G	0.20	2
63	RNF43	c.2156A>T	p.Y719F	0.10	2
64	PIK3CA	c.3140A>G	p.H1047R	6.40	1
64	TP53	c.637C>T	p.R213*	3.40	1
64	EPHA5	c.2737G>C	p.D913H	1.40	1
64	CCNE1	c.83C>G	p.S28C	0.70	1
64	TERT	c.-58-u5148C>A	.	0.60	1
65	EPHA3	c.2074+1G>C	.	9.50	1
65	PIK3CA	c.3140A>G	p.H1047R	7.70	1
65	INPP4B	c.2743C>G	p.P915A	5.90	1
65	MAP3K1	c.2384dupT	p.S796Vfs*11	6.10	1

65	APC	c.7403C>T	p.S2468L	7.40	1
65	FOXA1	c.798C>G	p.F266L	12.20	1
65	COL5A1	c.2770C>T	p.R924W	0.50	1
65	THOC1	c.1107delC	p.D370Mfs*9	1.10	1
66	PIK3CA	c.3140A>G	p.H1047R	0.90	2
66	RB1	c.2371A>T	p.K791*	0.70	2
66	TP53	c.644G>T	p.S215I	1.00	2
66	NF1	c.7907+2T>G	.	0.90	2
67	ABL1	c.1379G>A	p.R460H	8.40	1
67	NOTCH1	c.5598G>T	p.E1866D	7.30	1
67	NOTCH3	c.6385G>A	p.A2129T	3.80	1
67	ESR1	c.1613A>G	p.D538G	2.20	1
67	JAK2	c.1849G>T	p.V617F	1.30	1
67	SYK	c.1091A>G	p.K364R	0.50	1
68	MLL	c.2479T>A	p.F827I	0.50	3
68	CNOT4	c.1780A>T	p.T594S	0.40	3
69	PIK3CA	c.3132T>A	p.N1044K	20.30	3
69	CDK6	c.901C>T	p.Q301*	17.40	3
69	TP53	c.829T>G	p.C277G	14.50	3
69	PIK3CA	c.223C>G	p.Q75E	7.20	3
69	NOTCH4	c.5378G>C	p.R1793P	4.20	3
69	BRCA1	c.1068G>C	p.Q356H	3.30	3
69	ERBB2	c.3362C>T	p.S1121L	1.50	3
69	ERBB2	c.2840C>T	p.S947F	0.80	3
69	STAT1	c.2091delA	p.G698Efs*9	0.40	3
69	MTOR	c.7360T>A	p.S2454T	0.30	3
69	EPHA2	c.380A>T	p.D127V	0.30	3
69	ABCC11	c.1772A>C	p.N591T	0.30	3
70	ND			0.00	2
71	ATM	c.692A>G	p.H231R	45.40	1
71	TP53	c.1009C>T	p.R337C	36.20	1
71	NRAS	c.398T>G	p.L133R	30.00	1
71	ATR	c.3427G>A	p.V1143I	17.60	1
71	RNF43	c.473C>A	p.T158N	13.10	1
71	MLL4	c.6885C>A	p.Y2295*	11.70	1
71	AMOT	c.2405C>T	p.S802L	10.90	1
71	PIK3R1	c.1743G>C	p.L581F	8.80	1
71	MAP3K1	c.2816C>G	p.S939C	3.50	1
71	PTPRD	c.2716G>A	p.E906K	3.20	1

71	FLT4	c.1647C>G	p.F549L	3.10	1
71	NOTCH1	c.4756C>T	p.R1586C	2.90	1
71	MAPK1	c.243G>C	p.E81D	2.60	1
71	ACIN1	c.1267G>T	p.E423*	2.50	1
71	DDR2	c.2110C>G	p.L704V	2.20	1
71	ACIN1	c.3448G>T	p.E1150*	2.20	1
71	STK11IP	c.2884C>G	p.R962G	2.10	1
71	TERT	c.-58-u914G>C	.	2.00	1
71	CDK12	c.11C>T	p.S4L	1.90	1
71	PRKAA1	c.919C>A	p.P307T	1.50	1
71	PRKAA1	c.452C>G	p.S151C	1.40	1
71	RUNDC3A	c.439C>T	p.R147C	1.10	1
71	FCGR3A	c.91G>C	p.E31Q	0.90	1
71	TSC1	c.631G>C	p.E211Q	0.80	1
71	ERCC1	c.449C>G	p.P150R	0.70	1
71	MLL	c.8501C>A	p.S2834*	0.70	1
71	CBL	c.904C>A	p.Q302K	0.60	1
71	BRCA1	c.3848A>T	p.H1283L	0.50	1
72	PIK3CA	c.1035T>A	p.N345K	1.30	1
72	CDH1	c.1600G>T	p.E534*	0.80	1
73	PIK3CA	c.1035T>A	p.N345K	5.80	1
73	NOTCH1	c.5422G>A	p.D1808N	2.10	1
74	PIK3CA	c.1633G>A	p.E545K	1.70	1
74	TP53	c.574C>T	p.Q192*	1.10	1
74	CDH23	c.6511C>T	p.R2171C	0.70	1
75	TP53	c.490A>G	p.K164E	16.50	2
75	TRIO	c.2086G>A	p.D696N	13.30	2
75	PIK3CA	c.3140A>G	p.H1047R	5.50	2
75	ESR1	c.1138G>C	p.E380Q	2.60	2
75	MSH6	c.670G>C	p.E224Q	2.40	2
75	ESR1	c.1610A>C	p.Y537S	1.40	2
76	TP53	c.853G>A	p.E285K	17.64	3
76	PIK3CA	c.3140A>G	p.H1047R	15.97	3
76	RNF43	c.2005C[4>3]	p.Q670Rfs*30	10.92	3
76	JAK3	c.184+1_184+3delGTG	.	5.82	3
76	NOTCH2	c.7_8delGCinsTT	p.A3F	3.79	3
76	FLT3	c.20A>G	p.D7G	1.97	3
76	CDH23	c.7139C>T	p.P2380L	1.67	3
76	BRCA2	c.10103C>T	p.S3368F	1.03	3

76	DNMT3A	c.483G>A	p.M161I	1.09	3
76	TMEM247	c.460G>A	p.E154K	1.24	3
77	HLA-DRB1	c.398C>T	p.S133L	6.20	3
77	OR6F1	c.643T>C	p.F215L	2.00	3
77	TP53	c.672+2T>G	.	4.20	3
77	HSPD1	c.167G>A	p.G56E	2.64	3
78	FAT1	c.10660T>G	p.S3554A	2.21	2
78	GATA3	c.1322_1323insG	p.A442Rfs*66	10.49	2
78	ITM2A	c.331G>T	p.V111L	13.80	2
79	TP53	c.913A>T	p.K305*	47.81	2
79	PIK3CA	c.3140A>G	p.H1047R	34.97	2
79	PIK3CA	c.316G>A	p.G106S	28.91	2
79	RET	c.3251A>G	p.N1084S	28.76	2
79	ESR1	c.1610A>C	p.Y537S	16.69	2
79	POTEG	c.324C>G	p.C108W	9.68	2
79	HSPD1	c.167G>A	p.G56E	3.05	2
79	BRD4	c.56A>G	p.D19G	1.05	2
79	FCGR3A	c.194G>A	p.S65N	1.73	2
79	FGFR4	c.1162G>A	p.G388R	1.07	2
79	NOTCH4	c.370C>T	p.P124S	1.55	2
79	OR6F1	c.643T>C	p.F215L	1.09	2
80	THOC1	c.1120T>C	p.F374L	2.23	3
81	TP53	c.991C>T	p.Q331*	44.16	3
81	HSPD1	c.167G>A	p.G56E	5.22	3
81	ERBB2	c.2175G>C	p.L725F	1.85	3
81	ERBB2	c.2620G>A	p.D874N	2.03	3
81	MCL1	c.861C>G	p.I287M	2.63	3
81	PSTPIP1	c.211A>G	p.N71D	2.79	3
81	ZC3H13	c.239G>T	p.R80I	3.66	3
82	ND				3
83	DNMT3A	c.1445C>T	p.T482M	18.13	2
83	TP53	c.497C>G	p.S166*	20.00	2
83	HDAC4	c.2008G>A	p.G670R	12.46	2
83	JAK2	c.1018C>T	p.R340*	10.68	2
83	PIK3CA	c.3140A>G	p.H1047R	8.78	2
83	HSPD1	c.167G>A	p.G56E	3.40	2
83	AR	c.2191G>A	p.V731M	3.03	2
83	GPR114	c.65-2A>T	.	2.25	2
83	FGFR4	c.1162G>A	p.G388R	2.07	2

83	DNMT3A	c.1911+1G>A	.	1.35	2
83	FLCN	c.1202G>A	p.R401H	1.28	2
84	HSPD1	c.167G>A	p.G56E	2.56	2
84	PALB2	c.3256C>T	p.R1086*	1.80	2
84	ROS1	c.4738G>A	p.E1580K	1.55	2
85	HSPD1	c.167G>A	p.G56E	2.14	2
85	NOTCH3	c.6668C>T	p.A2223V	1.20	2
85	TMPRSS2	c.589G>A	p.V197M	1.52	2
86	PIK3CA	c.1624G>A	p.E542K	1.93	3
86	ERBB2	c.3020C>T	p.P1007L	1.31	3
87	ND				3
88	BRCA1	c.4388C>T	p.S1463F	14.47	3
88	SETD2	c.4855C>G	p.Q1619E	8.01	3
88	RYR3	c.14503G>C	p.E4835Q	6.85	3
88	MED12	c.5653G>A	p.V1885I	4.88	3
88	ERBB2	c.314G>C	p.G105A	4.15	3
88	TP53	c.711G>A	p.M237I	3.46	3
88	ERBB2	c.1848G>C	p.Q616H	2.51	3
88	ERBB2	c.3028G>A	p.G1010R	2.47	3
88	ERBB2	c.1606G>A	p.E536K	1.93	3
88	ERBB2	c.3250G>A	p.E1084K	1.59	3
88	WDR52	c.1409C>T	p.S470F	1.07	3
89	PIK3CA	c.1624G>A	p.E542K	5.24	2
89	BRCA2	c.1806A[8>7]	p.I605Yfs*9	2.90	2
90	FGFR4	c.1560G>C	p.E520D	12.83	2
90	NLRP4	c.2429G>A	p.R810H	26.48	2
90	TP53	c.920-12_923delCCTCTTTCCTAGCACT	.	22.13	2
90	ATM	c.6915G>C	p.Q2305H	14.19	2
90	PIK3CA	c.3049G>C	p.D1017H	14.04	2
90	PTEN	c.303C>G	p.I101M	13.00	2
90	ABCC8	c.2489C>T	p.S830F	11.88	2
90	ROBO3	c.2707G>A	p.A903T	11.47	2
90	HSPD1	c.167G>A	p.G56E	7.86	2
91	TP53	c.752T>G	p.I251S	7.31	2
91	TP53	c.743G>A	p.R248Q	19.12	2
91	HSPD1	c.167G>A	p.G56E	3.59	2
91	JAK2	c.172C>G	p.P58A	2.30	2
92	TP53	c.380C>A	p.S127Y	14.60	2

92	CDH23	c.7139C>T	p.P2380L	2.75	2
92	CDH23	c.6130G>A	p.E2044K	1.82	2
92	ERBB2	c.3418C>G	p.P1140A	1.55	2
92	BRCA1	c.3113A>G	p.E1038G	1.05	2
92	CSMD3	c.2362C>T	p.L788F	1.07	2
92	TMEM247	c.387_388insGAGCAG	p.R129_Q130insEQ	1.47	2
93	MSH6	c.116G>A	p.G39E	2.27	2
93	PIK3CA	c.3140A>T	p.H1047L	1.26	2
93	FAT1	c.10660T>G	p.S3554A	1.62	2
93	OR4A15	c.76C>T	p.P26S	1.05	2
94	TP53	c.747G>T	p.R249S	25.94	2
94	RICTOR	c.4883C>T	p.S1628L	11.57	2
94	RB1	c.2062C>G	p.L688V	6.61	2
94	CDH23	c.5023G>A	p.V1675I	2.04	2
94	CDH23	c.5996C>G	p.T1999S	1.48	2
94	EZH2	c.553G>C	p.D185H	1.24	2
94	FAT1	c.4985A>G	p.N1662S	2.55	2
94	FLT3	c.20A>G	p.D7G	1.87	2
94	OR2T4	c.757delA	p.I253Sfs*8	3.15	2
94	OR4A15	c.76C>T	p.P26S	1.40	2
94	OR4C6	c.398T>C	p.I133T	2.00	2
94	ROBO3	c.1247G>A	p.R416H	2.87	2
94	SETD2	c.3240G>A	p.M1080I	1.23	2
94	TMPRSS2	c.589G>A	p.V197M	1.35	2
95	RB1	c.1510C>T	p.Q504*	4.09	3
95	TP53	c.526T>A	p.C176S	5.57	3
95	HDAC4	c.1612G>A	p.E538K	2.41	3
95	MAP2K4	c.415G>T	p.E139*	2.28	3
95	FGFR1	c.160C>T	p.R54C	1.71	3
95	GNAS	c.152C>A	p.S51Y	1.24	3
95	IL7R	c.955G>T	p.E319*	1.13	3
95	OR5L2	c.831C>A	p.F277L	1.18	3
95	KEAP1	c.1067C>T	p.A356V	1.15	3
95	AKT2	c.141G>C	p.Q47H	1.08	3
95	FLCN	c.907G>A	p.G303R	1.00	3
96	KDR	c.889G>A	p.V297I	48.37	2
96	NOTCH3	c.6668C>T	p.A2223V	1.18	2
97	FMN2	c.4941G>A	p.M1647I	8.46	3

97	NOTCH4	c.731C>T	p.S244L	11.71	3
97	TP53	c.818G>A	p.R273H	27.27	3
98	TP53	c.708_709delCA	p.M237Vfs*2	6.00	2
98	FLT1	c.1A>G	p.0?	5.80	2
98	CDK12	c.4130C>T	p.S1377L	0.60	2
98	NOTCH1	c.6395C>T	p.T2132M	0.50	2
99	FGFR2	c.2315T>G	p.L772R	8.00	4
99	XBP1	c.319G>A	p.E107K	7.70	4
99	KDR	c.1699G>A	p.V567M	7.30	4
99	LRRC7	c.43C>T	p.R15*	6.70	4
99	TP53	c.818G>A	p.R273H	6.50	4
99	RICTOR	c.1252C>T	p.H418Y	5.20	4
100	TP53	c.889_890insC	p.H297Pfs*9	1.40	3

ND not detected

Supplementary Table S3. Somatic copy number variants (CNVs) identified in 100 plasma samples

Patient ID	Gene Symbol	CNV	Subtype (1=HR+/HER2-, 2=HR+/HER2+, 3=HR-/HER2+, 4=HR-/HER2-)
1	ERBB2	Copy number gain	3
1	CDK12	Copy number gain	1
2	FGFR1	Copy number gain	1
2	TOP1	Copy number gain	1
5	AURKA	Copy number gain	1
8	ERBB2	Copy number gain	2
8	FGFR1	Copy number gain	2
8	PIK3CA	Copy number gain	2
11	IGF1R	Copy number gain	1
11	FGFR1	Copy number gain	1
14	CDK12	Copy number gain	3
14	ERBB2	Copy number gain	3
46	FLT1	Copy number gain	1
46	RPS6KB1	Copy number gain	1
46	FGFR1	Copy number gain	1
46	AURKA	Copy number gain	1
53	IRS2	Copy number gain	1
60	FGFR1	Copy number gain	1
67	RNF43	Copy number gain	1
67	IGF1R	Copy number gain	1
67	RPS6KB1	Copy number gain	1
69	ERBB2	Copy number gain	3
69	CDK12	Copy number gain	3
69	MPL	Copy number gain	3
69	PTCH2	Copy number gain	3
75	ERBB2	Copy number gain	2
98	CDK12	copy number gain	2
98	ERBB2	copy number gain	2
